# Efficacy and safety of anlotinib combined with PD-1/PD-L1 inhibitors in malignant solid tumors: a meta-analysis and network meta-analysis

**DOI:** 10.3389/fimmu.2026.1780636

**Published:** 2026-03-24

**Authors:** Chen Wang, Ning Wang, Zijing Wu, Xinjuan Yu, Xiaolu Yu, Jing Wang, Jun Li, Yaozu Han

**Affiliations:** 1 School of Medicine and Pharmacy, Ocean University of China, Qingdao, China; 2 Department of Respiratory and Critical Care Medicine, Qingdao Municipal Hospital, University of Health and Rehabilitation Sciences, Qingdao, China; 3 Clinical Research Center, Qingdao Key Laboratory of Common Diseases, Qingdao Municipal Hospital, University of Health and Rehabilitation Sciences, Qingdao, China

**Keywords:** anlotinib, malignant solid tumors, meta-analysis, PD-1 inhibitors, PD-L1 inhibitors

## Abstract

**Objective:**

To evaluate the efficacy and safety of anlotinib combined with programmed cell death protein 1/programmed death-ligand 1 (PD-1/PD-L1) inhibitors in the treatment of malignant solid tumors.

**Methods:**

PubMed, Web of Science, Embase, and Cochrane Library databases were searched to collect randomized controlled trials (RCTs), cohort studies, and single-arm studies comparing anlotinib combined with PD-1/PD-L1 inhibitors (combination group) against other therapies (control group) for the treatment of malignant solid tumors. The search period spanned from the inception of each database to November 2025.

**Results:**

26 studies involving 3,263 patients were identified. The Objective Response Rate (ORR) in the combination group was 57% (95% CI: 47%-67%), with a Disease Control Rate (DCR) of 90% (95% CI: 84%-93%). The incidence of adverse reactions included fatigue (RR = 1.196, 95% CI: 1.024-1.396) and hypoalbuminemia (RR = 1.336, 95% CI: 1.121-1.593). Compared with the control group, the combination group significantly improved ORR in malignant solid tumors (RR = 1.70, 95% CI: 1.29-2.25, P ≤ 0.0001), DCR (RR = 1.08, 95% CI: 1.02-1.14, P = 0.009), Overall Survival (OS) (HR = 0.64, 95% CI: 0.54-0.76, P ≤ 0.001), and Progression Free Survival (PFS) (HR = 0.48, 95% CI: 0.39-0.59, P ≤ 0.001). The incidence of hypoalbuminemia was higher in the combination group than in the control group. Results from a network meta-analysis indicated superior efficacy for the combination of anlotinib and TQB2450.

**Conclusion:**

Anlotinib combined with PD-1/PD-L1 inhibitors demonstrates significant efficacy in treating multiple tumor types. The combination of anlotinib and TQB2450 offers advantages, but attention should be paid to the occurrence of hypoproteinemia.

## 1 Introduction

Malignant solid tumors account for a disproportionately high number of deaths among all cancer-related fatalities worldwide (1). Despite advances in targeted therapy and immunotherapy, monotherapy regimens often yield limited clinical benefit. Conventional chemotherapy exhibits an objective response rate (ORR) below 20% and is frequently associated with drug resistance (2). immune checkpoint inhibitors (ICIs), such as programmed cell death protein 1/programmed death-ligand 1 (PD-1/PD-L1) inhibitors, are constrained by tumor microenvironment (TME) heterogeneity, including immune-suppressive cell infiltration, aberrant vasculature, and hypoxia, resulting in long-term responses in only a subset of patients (3). Consequently, combination strategies with synergistic mechanisms have become a pivotal direction in oncology.

As a multi-target tyrosine kinase inhibitor, anlotinib has been approved for the treatment of lung cancer, soft tissue sarcoma, thyroid cancer, and other malignancies. However, in clinical practice, the efficacy of anlotinib monotherapy in other cancer types remains inconclusive, with suboptimal responses in some patients and notable interindividual variability, which somewhat restricts its clinical utility (4–6). The combination of anlotinib with PD-1/PD-L1 inhibitors has emerged as a promising approach to overcome resistance and enhance therapeutic outcomes. In this study, we systematically evaluate the efficacy and safety of anlotinib combined with PD-1/PD-L1 inhibitors in the treatment of malignant solid tumors through a meta-analysis.

## 2 Materials and methods

### 2.1 Data sources and search strategy

This meta-analysis was conducted in accordance with a pre-established protocol and reported following the Preferred Reporting Items for Systematic Reviews and Meta-Analyses (PRISMA) guidelines (7). The study protocol was registered *a priori* in the PROSPERO database (registration number: CRD420251186088 https://www.crd.york.ac.uk/PROSPERO/view/CRD420251186088). A systematic search was performed across multiple electronic databases, including PubMed, Embase, Web of Science and Cochrane Library, from their inception until November 2025. The search strategy combined Medical Subject Headings (MeSH) with free-text terms, incorporating key concepts such as “anlotinib”, “PD-1 inhibitor”, “PD-L1 inhibitor”, and “randomized controlled trial”. The detailed search strategies are provided in **Supplementary Table 1**.

### 2.2 Study selection and data extraction

The literature was independently screened by two researchers based on inclusion and exclusion criteria. In case of any disagreement, a third researcher would make a decision through discussion. Data extraction included: first author, publication year, tumor type, study type, sample size, interventions, median follow-up time, outcome indicators, and so on.

Primary outcome measures comprised: ORR and Disease Control Rate (DCR). Secondary outcome measures included: Progression-Free Survival (PFS), Overall Survival (OS), and treatment-related adverse events (TRAEs).

### 2.3 Inclusion and exclusion criteria

The study types covered in this research include randomized controlled trials (RCTs), cohort studies, and single-arm trials (SATs). Inclusion criteria: (i) Patients with histologically or cytologically confirmed locally advanced or advanced malignant tumors, without restrictions on gender, region, ethnicity, tumor type, or specific tumor status. (ii) Interventions include oral anlotinib administration and intravenous PD-1/PD-L1 inhibitor administration (combination group). (iii) PD-1/PD-L1 inhibitors included TQB2450, camrelizumab, durvalumab, penpulimab, sintilimab, and pembrolizumab.

Exclusion criteria for this study’s literature were: (i) duplicated publications; (ii) meta-analyses, systematic reviews, and animal studies; (iii) studies with inconsistent research content; (iv) studies with inconsistent interventions, inconsistent experimental designs, lacking full text, or unable to extract primary outcome measures.

### 2.4 Quality assessment

The methodological quality of the included RCTs was assessed using the Cochrane Risk of Bias tool (RoB 1.0), as described in the Cochrane Handbook for Systematic Reviews of Interventions (version 5.1.0). This tool evaluates key domains, including random sequence generation, allocation concealment, blinding of participants and personnel, blinding of outcome assessment, incomplete outcome data, selective reporting, and other potential sources of bias. Each domain was judged as having a “low risk,” “high risk,” or “unclear risk” of bias.

In contrast, the quality of single-arm trials and cohort studies was evaluated using the Newcastle-Ottawa Scale (NOS). This tool assigns a score ranging from 0-9, where a score of 0–3 indicates poor quality, 4–6 indicates fair quality, and 7–9 indicates good quality. Any discrepancies in the ratings were resolved through discussion to reach a consensus.

### 2.5 Statistical analysis

Meta-analysis was conducted using RevMan 5.3, Stata 14.0, and R-4.4.2 software. Binary variables were expressed as relative risk ratios (RR) with 95% confidence intervals (CI). Survival data analysis utilized hazard ratios (HR) with 95% CI. Statistical heterogeneity among studies was assessed using Q-tests and I² tests. When P ≥ 0.1 and I² < 50%, indicating no statistical heterogeneity among studies, a fixed-effect model was used; otherwise, a random-effects model was applied. Sources of heterogeneity were explored through sensitivity and subgroup analyses. Sensitivity analysis employed the Leave-One-Out method; publication bias was assessed using funnel plots and Egger’s test. The significance level was set at α = 0.05. Network meta-analysis was conducted using Stata 14.0 software, with all outcome measures analyzed using random-effects models. Evidence networks illustrated relationships among interventions, where nodes represented different interventions and edges depicted direct comparisons between interventions in included studies. The weight of each intervention was determined by the number of studies containing direct comparisons. Node size reflected the sample size of the intervention, and line thickness represented the number of pairwise comparisons. When closed loops existed in the evidence network, inconsistency tests were performed. If P > 0.05, no inconsistency was present, and the consistency model was used for analysis. The Surface Under the Cumulative Ranking Curve (SUCRA) was used to rank the effectiveness of different interventions, with higher SUCRA values indicating superior efficacy. A comparison-adjusted funnel plot was constructed to assess publication bias among the included studies.

## 3 Results

### 3.1 Study eligibility screening and baseline features

**Figure 1:**
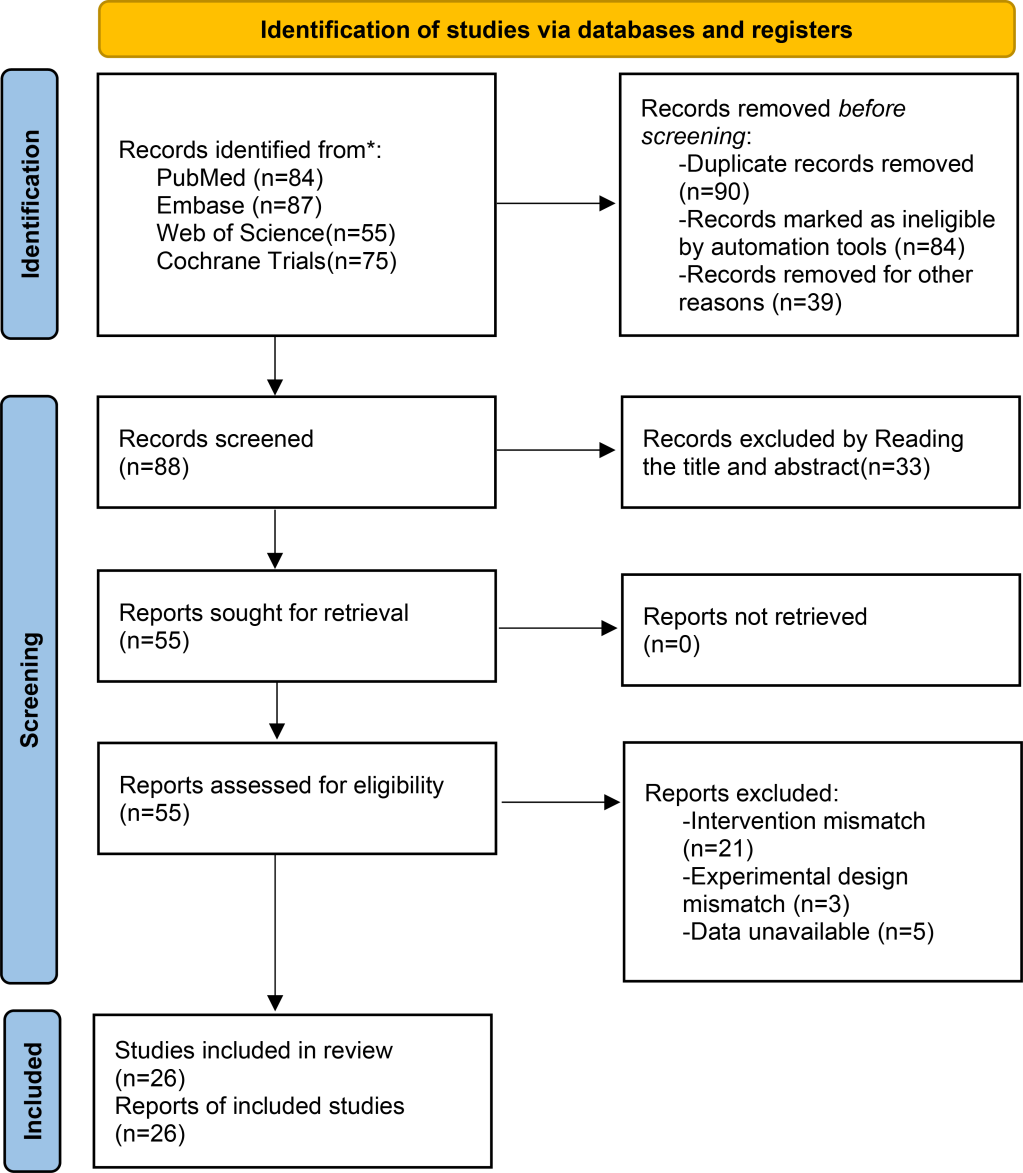
Literature search flow chart.

A total of 301 articles were initially retrieved through database searches including PubMed, Embase, Web of Science, and the Cochrane Library. After initial screening, 213 articles were excluded. Subsequently, following a detailed assessment of titles and abstracts, and the exclusion of studies due to intervention mismatch, experimental design mismatch, or unavailable data, 26 studies (8–33) were included in the final analysis, comprising 14 RCTs (8–10, 12, 13, 17, 21–24, 28, 30, 32, 33), nine single-arm trials (11, 14–16, 20, 25–27, 31), and three cohort studies (18, 19, 29), involving a total of 3,263 patients (**Figure 1**). The included studies covered a range of tumor types: non-small cell lung cancer (NSCLC, n=8), small cell lung cancer (SCLC, n=5), esophageal squamous cell carcinoma (ESCC, n=4), renal cell carcinoma (RCC, n=2), biliary tract cancer (BTC, n=2), hepatocellular carcinoma (HCC, n=2), endometrial cancer (EC, n=1), cervical cancer (CC, n=1), and ovarian cancer (OC, n=1) (**Table 1**).

**Table 1:** Basic characteristics of the literature.

Study	Tumor type	Study design	Sample size		Intervention		mFU (years)	Outcome
			Joint/control	Joint group		Control group		
W. Zhang et al. 2023 (30)	relapsed/refractory NSCLC	RCT Phase Ib	68/33	ANTN 10/12mg+TQB2450 1200mg		TQB24501200mg+ Placebo	14.5	ORR;DCR; TRAEs
Y. Cheng et al. 2024 (9)	treatment-naïve SCLC	RCT Phase III	246/247	ANTN 12mg+TQB2450 1200mg+Chemotherapy		Placebo + Chemotherapy	14	ORR;DCR; PFS;OS;TRAEs
X. Sheng et al. 2024 (21)	treatment-naïve RCC	RCT Phase III	266/265	ANTN 12mg+TQB2450 1200mg		Sunitinib 50mg	19.52	ORR;PFS; OS
C. Zhang et al. 2024 (28)	relapsed/refractory NSCLC	RCT	27/23	ANTN 12mg+TQB2450 1200mg		TQB2450 1200mg+Placebo	15.1	PFS
X. Chen et al. 2025 (8)	treatment-naïve EC	RCT Phase II	38/33	ANTN 8mg+TQB2450 1200mg+Chemotherapy		TQB2450 1200mg+ Chemotherapy	16.2	ORR;PFS; OS;TRAEs
B. Han et al. 2025 (12)	treatment-naïve NSCLC	RCT Phase III	354/177	ANTN 12mg+TQB2450 1200mg		PEMBRO 200mg+ Placebo	11.4	ORR;PFS
A. Zhou et al. 2025 (32)	treatment-naïve RCC	RCT Phase III	264/263	ANTN 12mg+TQB2450 1200mg		Sunitinib 50mg	22.8	ORR;DCR; PFS;TRAEs
Y. Wang et al. 2021 (23)	relapsed/refractory NSCLC	RCT Phase III	44/44	ANTN 12mg+CAM 200mg		ANTN 12mg	/	ORR;DCR
M. Xu et al. 2025 (24)	treatment‐naïve ESCC	RCT Phase II	30/30	ANTN 8mg+CAM 200mg+Chemotherapy		CAM 200mg+ Chemotherapy	14.5	ORR;DCR; PFS;OS;TRAEs
B. Han et al. 2024 (13)	treatment‐naïve SCLC	RCT Phase III	34/32	ANTN 12mg+DURV 1500mg		DURV 1500mg	15.1	PFS;OS
C. Wang et al. 2023 (22)	treatment‐naïve NSCLC	RCT Phase II	16/16	ANTN 12mg+PEN 200mg		PEN 200mg	5.3	ORR
T. Chu et al. 2024 (10)	treatment‐naïve NSCLC	RCT	14/16	ANTN 12mg+SIN 200mg		NIVO 360mg/TOR 240mg/TIS 200mg+ Chemotherapy	/	ORR;DCR; TRAEs
J. Li et al. 2025 (17)	treatment‐naïve BTC	RCT Phase II	40/40	ANTN 10mg+SIN 200mg+Chemotherapy		Chemotherapy	13.4	ORR;DCR; PFS;OS;TRAEs
J. Zhou et al. 2025 (33)	relapsed/refractory NSCLC	RCT	59/59	ANTN 12mg+SIN 200mg		SIN 200mg	/	ORR;DCR
B. Han et al. 2021 (11)	treatment‐naïve NSCLC	SAT	22	ANTN 12mg +SIN 200mg			15.8	ORR;DCR
C. Han et al. 2022 (14)	treatment‐naïve HCC	SAT Phase Ib/II	31	ANTN 8mg+PEN 200mg			23	ORR;DCR
C. Lan et al. 2022 (16)	relapsed/refractory OC	SAT Phase Ib	34	ANTN 12mg+TQB2450 1200mg			8.6	ORR;DCR; TRAEs
Q. Xu et al. 2022 (25)	relapsed/refractory CC	SAT Phase II	42	ANTN 10mg+SIN 200mg			13	ORR;DCR
S. Jin et al. 2023 (15)	relapsed/refractory BTC	SAT Phase II	20	ANTN 12mg+SIN 200mg			12.5	ORR;DCR; TRAEs
N. Yang et al. 2023 (26)	relapsed/refractory SCLC	SAT Phase II	21	ANTN 10mg+PEN 200mg			/	ORR;DCR
Y. Zhang et al. 2023 (31)	relapsed/refractory SCLC	SAT Phase II	38	ANTN 10mg+PEN 200mg			/	ORR;DCR
H. Yuan et al. 2025 (27)	treatment‐naïve HCC	SAT Phase II	31	ANTN 12mg+TQB2450 1200mg+DEB-TACE			/	ORR;DCR; TRAEs
X. Meng et al. 2025 (20)	treatment‐naïve ESCC	SAT Phase II	46	ANTN 12mg+TQB2450 1200mg			17.64	ORR;DCR; TRAEs
Y. Liu et al. 2022 (18)	relapsed/refractory ESCC	Cohort	48/50	ANTN 12mg+TOR 240mg/CAM 200mg/SIN 200mg/PEMBRO 200mg		ANTN 12mg	9.3	ORR;DCR; TRAEs
W. Zhang et al. 2024 (29)	relapsed/refractory ESCC	Cohort	32/26	ANTN 12mg+CAM 200mg		ANTN 12mg+S-1 50mg	25.75	ORR;DCR; TRAEs
S. Ma et al. 2024 (19)	relapsed/refractory SCLC	Cohort	42	ANTN 12mg+SIN 200mg			24.8	ORR;DCR; TRAEs

NSCLC, non-small cell lung cancer; SCLC, small cell lung cancer; ESCC, esophageal squamous cell carcinoma; RCC, renal cell carcinoma; BTC, biliary tract cancer; HCC, hepatocellular carcinoma; EC, endometrial cancer; CC, cervical cancer; OC, ovarian cancer; RCT, randomized controlled trials; SAT, single-arm trials; ANTN, anlotinib; TQB2450, benmelstobart; SU, sunitinib; PEMBRO, pembrolizumab; CAM, camrelizumab; DURV, durvalumab; PEN, penpulimab; SIN, sintilimab; TOR, toripalimab; NIVO, nivolumab; TIS, tislelizumab; S-1, tegafur/gimeracil/oteracil potassium, an oral fluoropyrimidine anticancer agent; mFU, median follow-up; ORR, Objective Response Rate; DCR, Disease Control Rate; PFS, Progression-Free Survival; OS, Overall Survival; TRAEs, treatment-related adverse events.

### 3.2 Risk of bias in the inclusion literature

All 14 RCTs were judged to be at low risk for other potential sources of bias, although only two RCTs described adequate allocation concealment and 10 RCTs did not employ blinding procedures. For single-arm and retrospective studies, quality was assessed using the NOS, with scores ranging from five to eight, indicating fair to good quality. The detailed results of the quality assessment are presented in **Supplementary Figure 1**, **Supplementary Table 2**.

### 3.3 Results of meta-analysis

#### 3.3.1 ORR

**Figure 2:**
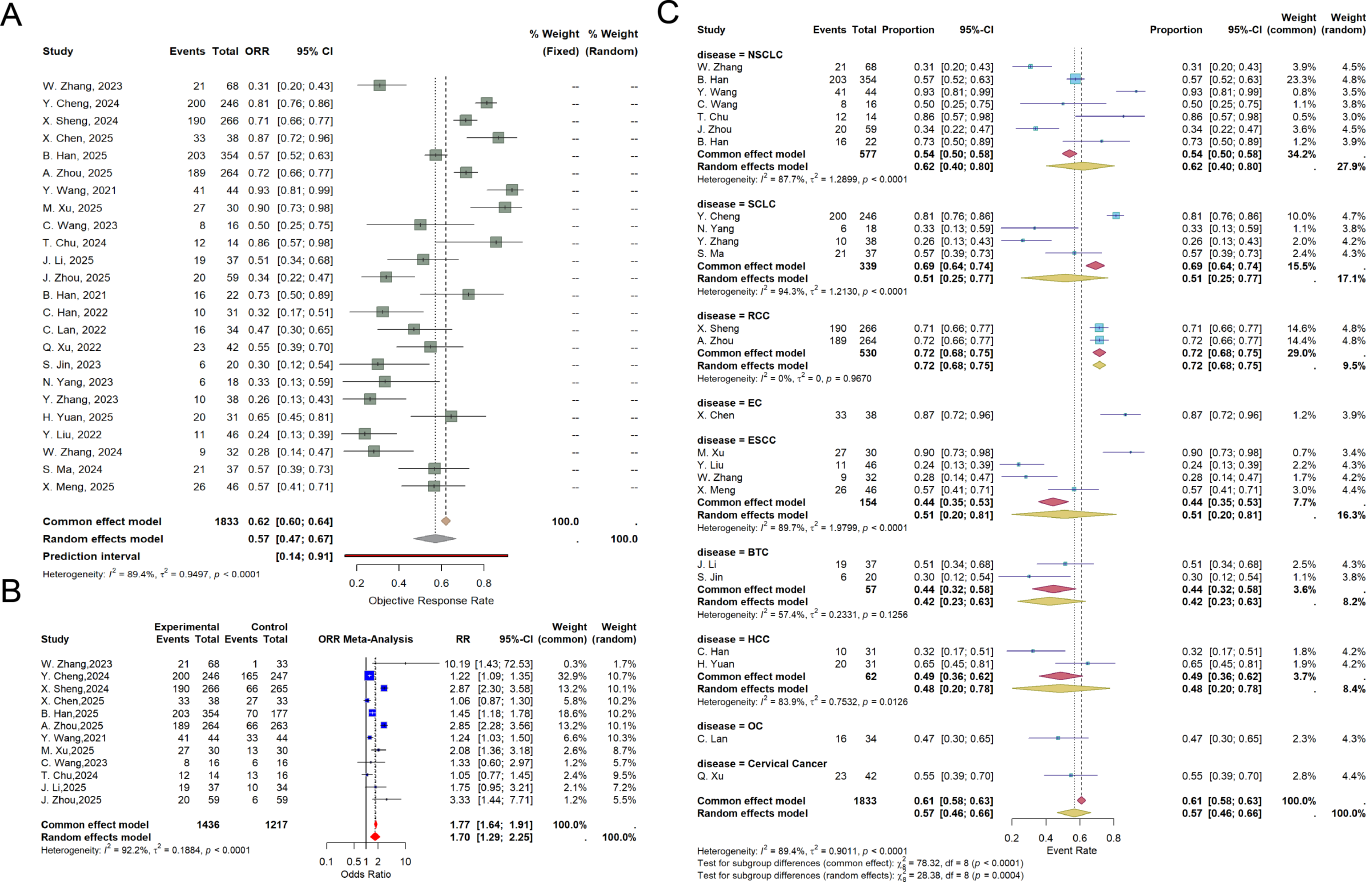
**(A)** Forest plot of ORR, showing pooled rates with 95% CIs for anlotinib plus PD−1/PD−L1 inhibitors in a meta−analysis of RCTs and non−RCTs; **(B)** Forest plot of ORR in RCTs, showing the pooled RR with 95% CIs for anlotinib plus PD-1/PD-L1 inhibitors versus control; **(C)** Forest plot of ORR subgroup analysis, showing the pooled rates with 95% CIs for each subgroup.

The ORR was reported in 24 studies. Significant heterogeneity was observed among these studies (P < 0.001, I² = 89.4%), and a random-effects model was therefore applied for the meta-analysis. The pooled analysis demonstrated an ORR of 57% (95% CI: 47%-67%) in the combination group (**Figure 2A**).

A separate analysis of 12 RCTs also revealed significant heterogeneity (P < 0.001, I² = 92.2%), leading to the use of a random-effects model. The results indicated that the combination group had a significantly higher ORR compared to the control group (RR = 1.70, 95% CI: 1.29-2.25) (**Figure 2B**).

Subgroup analysis by tumor type demonstrated the following pooled ORR for the combination group: NSCLC: 62% (95% CI: 40%-80%), SCLC: 51% (95% CI: 25%-77%), RCC: 72% (95% CI: 68%-75%), ESCC: 51% (95% CI: 20%-81%), BTC: 42% (95% CI: 23%-63%), and HCC: 48% (95% CI: 20%-78%) (**Figure 2C**). Subgroup analyses of prior treatment status in RCTs and non-RCTs showed that, based on pooled ORR results, the combination of anlotinib plus PD-1/PD-L1 inhibitors was more effective in treatment-naïve patients than in those with relapsed/refractory disease (68% *vs* 42%) (**Supplementary Figure 2A**). Subgroup analysis based on the inclusion or exclusion of chemotherapy in the combination regimen revealed that, according to pooled ORR, the addition of chemotherapy to anlotinib plus PD-1/PD-L1 inhibitors yielded better efficacy (79% *vs* 51%) (**Supplementary Figure 2B**).

#### 3.3.2 DCR

**Figure 3:**
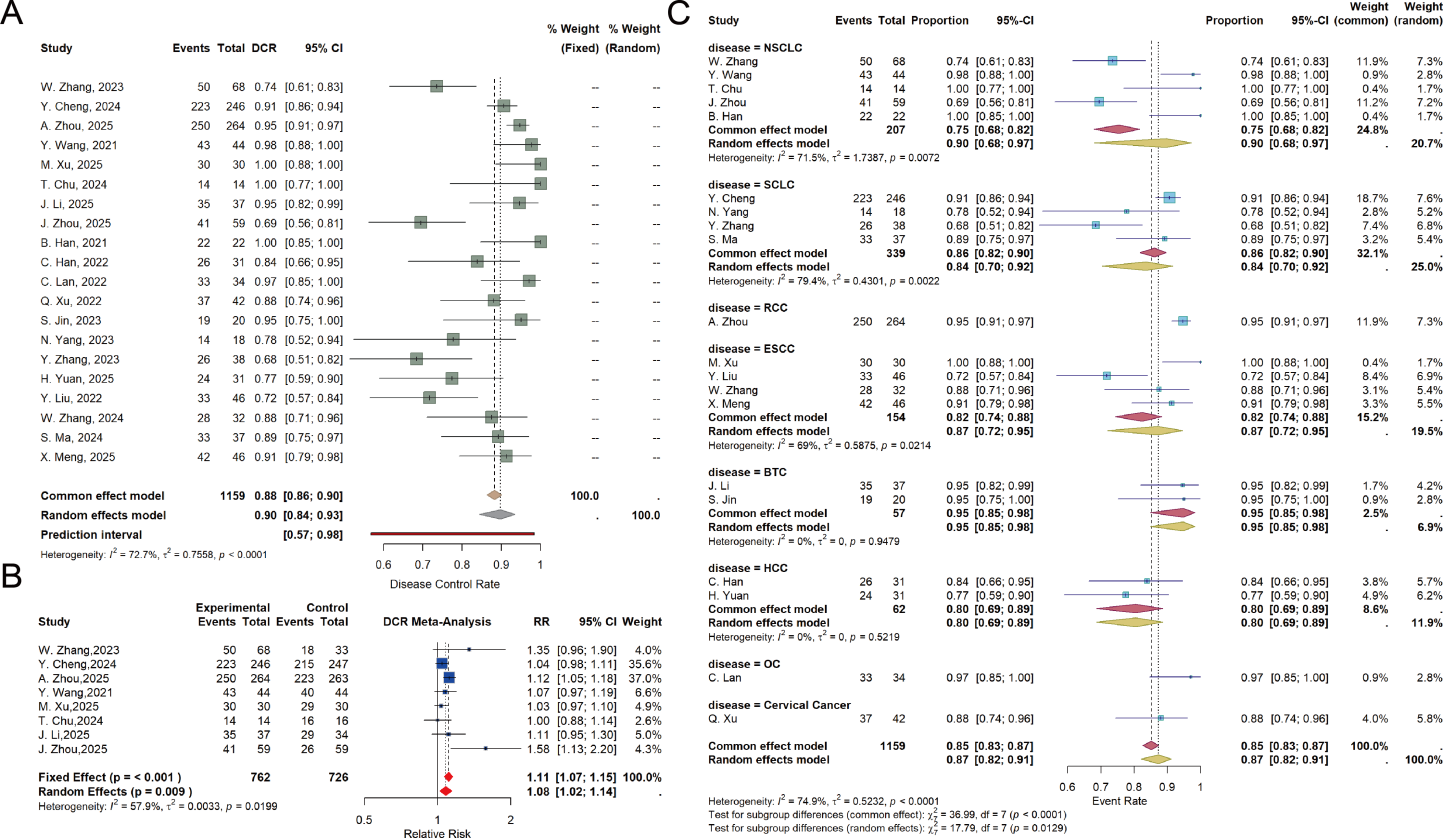
**(A)** Forest plot of DCR, showing pooled rates with 95% CIs for anlotinib plus PD−1/PD−L1 inhibitors in a meta−analysis of RCTs and non−RCTs; **(B)** Forest plot of DCR in RCTs, showing the pooled RR with 95% CIs for anlotinib plus PD-1/PD-L1 inhibitors versus control; **(C)** Forest plot of DCR subgroup analysis, showing the pooled rates with 95% CIs for each subgroup.

The DCR was reported in 20 studies. Significant heterogeneity was observed among these studies (P< 0.001, I²= 72.7%), and a random-effects model was therefore applied. The pooled analysis demonstrated a DCR of 90% (95% CI: 84%-93%) in the combination group (**Figure 3A**). Analysis of eight RCTs indicated that the DCR in the combination group was comparable to that of the control group (RR = 1.08, 95% CI: 1.02-1.14) (**Figure 3B**).

Subgroup analysis based on tumor type revealed the following DCR estimates for the combination group: 90% (95% CI: 68%-97%) for NSCLC, 84% (95% CI: 70%-92%) for SCLC, 87% (95% CI: 72%-95%) for ESCC, 0.95 (95% CI: 85%-98%) for BTC, and 80% (95% CI: 69%-89%) for HCC(**Figure 3C**). Subgroup analyses of prior treatment status in RCTs and non-RCTs showed that, based on pooled DCR results, the combination of anlotinib plus PD-1/PD-L1 inhibitors was more effective in treatment−naïve patients than in those with relapsed/refractory disease (91% *vs* 83%) (**Supplementary Figure 3A**). Subgroup analysis based on the inclusion or exclusion of chemotherapy in the combination regimen revealed that, according to pooled DCR, the addition of chemotherapy to anlotinib plus PD-1/PD-L1 inhibitors yielded better efficacy (91% *vs* 87%) (**Supplementary Figure 3B**).

#### 3.3.3 Survival data analysis

**Figure 4:**
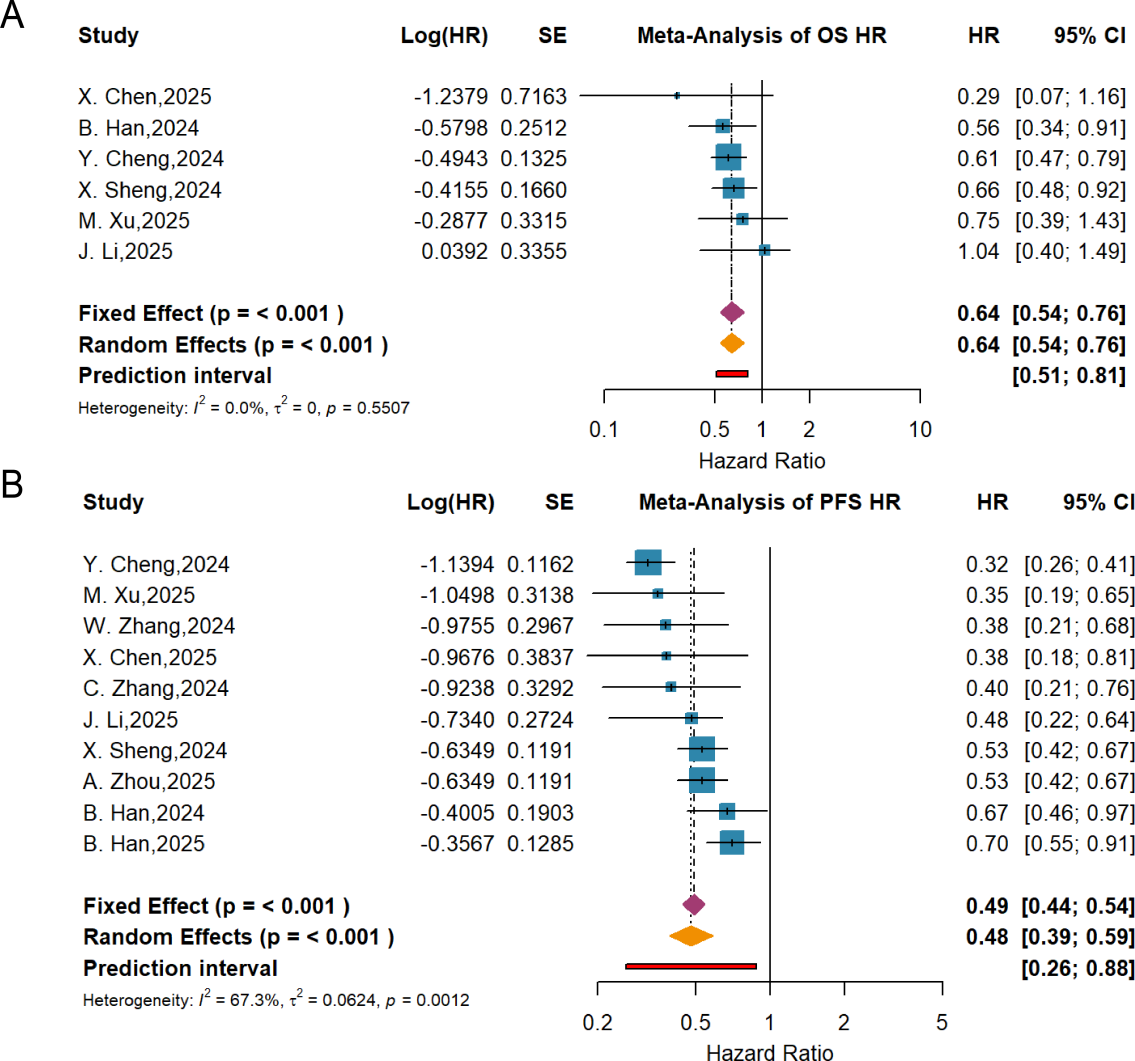
Forest plot of OS **(A)** and PFS **(B)** from RCTs, showing the pooled HRs with 95% CIs for anlotinib plus PD-1/PD-L1 inhibitors versus control.

Pooled analysis of OS data from six RCTs, performed using a fixed-effects model (P = 0.5507, I² = 0.0%), demonstrated that the combination therapy significantly improved OS in cancer patients (HR = 0.64, 95% CI: 0.54-0.76) (**Figure 4A**). For PFS, meta-analysis of 10 RCTs employing a random-effects model (P = 0.0012, I² = 67.3%) showed that the combination group also achieved significant improvement in PFS (HR = 0.48, 95% CI: 0.39-0.59) (**Figure 4B**).

#### 3.3.4 TRAEs

Among TRAEs evaluated in RCTs, only two demonstrated a statistically significant increased risk in the combination group compared to the control group: fatigue (RR = 1.196, 95% CI: 1.024-1.396) and hypoalbuminemia (RR = 1.336, 95% CI: 1.121-1.593). No significant differences in risk were observed for other TRAEs between the two groups (**Table 2**). However, a meta-analysis combining RCTs and non-RCTs indicated that the overall incidence range for these TRAEs was 18% to 41%, suggesting a low actual occurrence rate and overall manageable safety profile (**Supplementary Table 3**).

**Table 2:** Meta-analysis results of TRAEs in RCTs.

Adverse events	Study	Heterogeneity		RR (95%CI)	P
		P	I^2^		
Anemia	6	P < 0.001	93.30	0.701 (0.452-1.087)	0.113
Hypertension	6	P < 0.001	83.70	1.995 (0.948-4.198)	0.069
Reduced platelet count	6	P < 0.001	97.10	0.911 (0.417-1.991)	0.750
AST elevation	5	0.018	66.60	1.141 (0.7778-1.674)	0.499
Fatigue	5	0.297	18.50	1.196 (1.024-1.396)	0.024
Hand-foot skin reaction	5	P < 0.001	82.30	2.397 (0.525-10.939)	0.259
Proteinuria	5	0.003	74.90	1.706 (0.974-2.989)	0.062
Diarrhea	5	0.011	69.40	1.738 (0.829-3.646)	0.143
Hypoalbuminemia	5	0.249	25.90	1.336 (1.121-1.593)	0.001
Reduced neutrophil	5	P < 0.001	97.70	0.944 (0.357-2.500)	0.908
Nausea	5	0.123	44.80	0.868 (0.728-1.035)	0.114

### 3.4 Network meta-analysis

**Figure 5:**
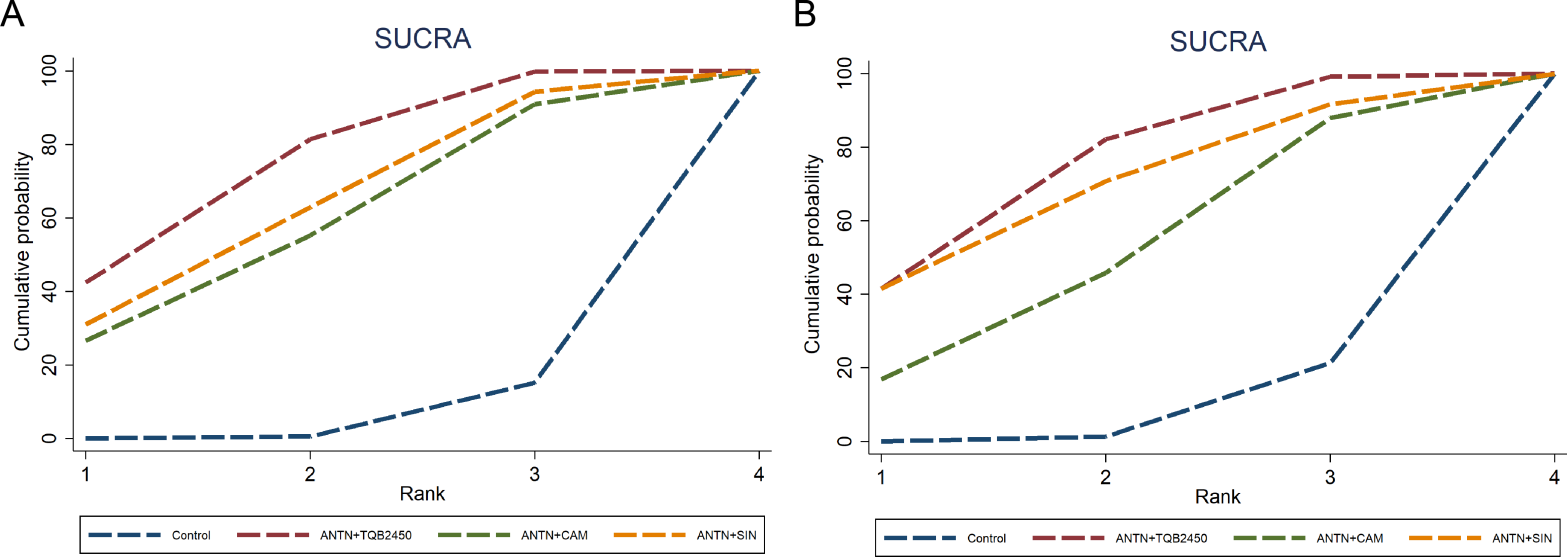
SUCAR plot for ORR **(A)** and DCR **(B)**. SUCRA provides a numerical summary of the rank distribution for each treatment, with values ranging from 0% to 100%; higher values indicate a greater likelihood of being among the top-ranked treatments.

A network meta-analysis revealed no closed loops among the interventions. The network plot is shown in **Supplementary Figures 4A, B**, analyzed using a consistency model. The ORR and DCR of anlotinib combined with TQB2450, camrelizumab, and sintilimab were compared. The network meta-analysis forest plot and league table showed that anlotinib combined with TQB2450 had a higher combined ORR (RR = 1.83, 95% CI 1.21–2.76) than the other two combination regimens, while no significant difference was observed in the combined DCR results (**Supplementary Figures 4C, D**; **Supplementary Tables 4**, **5**). For ORR and DCR, the SUCRA plot demonstrated the largest area under the curve for the anlotinib plus TQB2450 regimen, confirming its superior efficacy advantage over anlotinib combined with either camrelizumab or sintilimab (**Figure 5**).

### 3.5 Bias test and sensitivity analysis

A leave-one-out sensitivity analysis was performed on the pooled estimates for ORR (from 24 studies and 12 RCTs) and DCR (from 20 studies and eight RCTs), which demonstrated no substantial changes in the effect sizes after sequentially excluding each study, indicating the robustness of the findings. The detailed results of the sensitivity analysis are presented in **Supplementary Figures 5**, **6**.

**Figure 6:**
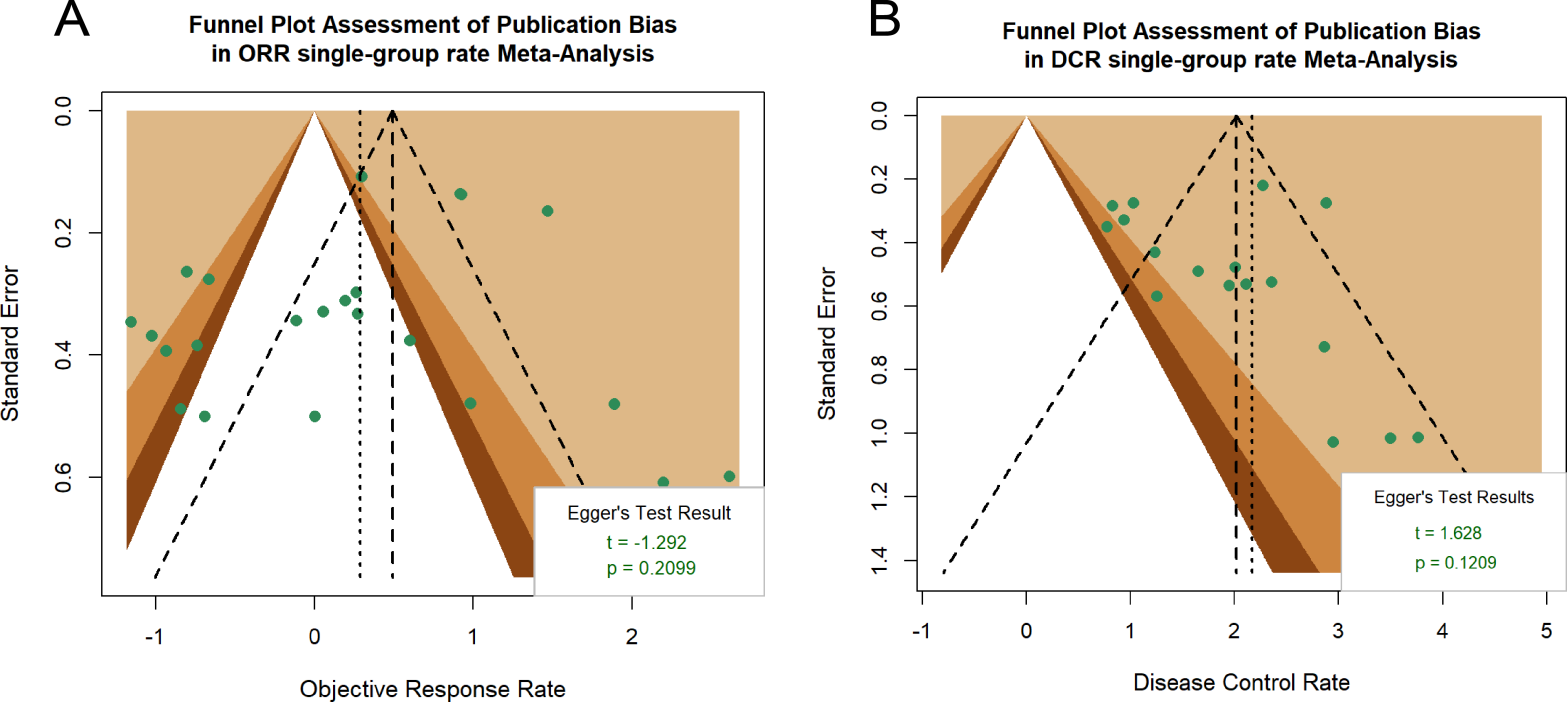
The funnel plot for publication bias of ORR **(A)** and DCR **(B)**. Egger’s test was performed to statistically evaluate funnel plot asymmetry.

For the primary outcome measures of ORR and DCR in RCTs and non-RCTs, funnel plots were constructed to assess potential publication bias. Visual inspection revealed near-symmetrical distribution of study points for both outcomes. Furthermore, Egger’s regression test showed no significant evidence of publication bias (ORR P = 0.2099; DCR P = 0.1209). The integrated results suggest a low likelihood of substantial publication bias in the ORR and DCR analyses (**Figures 6A, B**). The publication bias funnel plot for RCTs is shown in **Supplementary Figure 7**.

A funnel plot was constructed to compare outcome measures across studies included in the network meta-analysis. The results revealed an asymmetric scatter distribution, with some study points lying outside the 95% CI range. This suggests potential publication bias or small-sample effects (**Supplementary Figure 8**).

## 4 Discussion

This study represents the first systematic review and meta-analysis to comprehensively evaluate the efficacy and safety of anlotinib combined with PD-1/PD-L1 inhibitors (TQB2450, camrelizumab, durvalumab, penpulimab, and sintilimab) across different cancer types (NSCLC, SCLC, ESCC, RCC, BTC, HCC, EC, CC, and OC). It also compares the therapeutic outcomes of anlotinib in combination with TQB2450, camrelizumab, and sintilimab. The control groups received standard antitumor regimens, including chemotherapy alone, targeted therapy (e.g., sunitinib or anlotinib monotherapy), immunotherapy (e.g., PD-1/PD-L1 inhibitor monotherapy), combination therapy (e.g., immunotherapy plus chemotherapy, or targeted therapy plus chemotherapy), or placebo-controlled treatments. Pooled analysis demonstrated that, compared with these standard regimens alone, the combination of anlotinib with PD-1/PD-L1 inhibitors conferred significant advantages across multiple efficacy endpoints. For ORR and DCR, the combination of anlotinib and PD-1/PD-L1 inhibitors demonstrated better efficacy in treatment-naïve patients, and the addition of chemotherapy to the regimen yielded further improved outcomes. Notably, the combination of anlotinib and TQB2450 exhibited the most pronounced efficacy.

The advent of ICIs has brought breakthrough progress in cancer treatment; however, their clinical application still faces significant challenges. Extensive research indicates that, despite the remarkable potential of immunotherapy, a considerable proportion of patients experience primary or acquired resistance. The core of this resistance mechanism lies in the immunosuppressive nature of the TME and the resulting impairment of immune cell infiltration (34). Particularly in “immune-cold” tumors, characterized by a lack of T-cell infiltration, enrichment of immunosuppressive cells (such as regulatory T cells), and a physiological barrier formed by various immunosuppressive molecules, PD-1/PD-L1 inhibitor monotherapy often fails to elicit an effective antitumor immune response (35, 36). This resistance is primarily attributed to two key factors: insufficient infiltration of CD8^+^ effector T cells into the tumor tissue and an increased proportion of regulatory T cells (Tregs) within the TME. This imbalance in cellular composition further reinforces the immunosuppressive state, impairs T-cell activation and migration to the tumor site, and ultimately leads to a non-inflammatory T-cell profile, resulting in the failure of antitumor immune responses.

Anlotinib is a novel oral small-molecule multi-target tyrosine kinase inhibitor that precisely targets key receptors involved in tumor progression, including vascular endothelial growth factor receptors (VEGFR1, VEGFR2/KDR, VEGFR3), stem cell factor receptor (c-Kit), platelet-derived growth factor receptor α (PDGFR-α), and fibroblast growth factor receptors (FGFR1, FGFR2, FGFR3) (37). Its core mechanism involves dual inhibition: on one hand, it blocks angiogenesis-related signaling pathways, thereby suppressing the formation of tumor vasculature and cutting off nutrient supply to the tumor; on the other hand, it directly inhibits proliferation-related kinases within tumor cells, curbing their growth and dissemination (38). Compared with traditional tyrosine kinase inhibitors, anlotinib exhibits a broader spectrum of target coverage and a more comprehensive inhibitory profile (39). However, similar to most anti-angiogenic agents, anlotinib monotherapy has significant limitations. Research data indicate that for NSCLC treated with anlotinib alone, the median Overall Survival (mOS) was 9.6 months, the median Progression-Free Survival (mPFS) was 5.4 months, and the ORR was only 9.2% (40). These data suggest that while anlotinib possesses certain antitumor activity, its efficacy as a single agent is limited. The underlying reason for this limitation lies in the ability of tumor cells to develop resistance through mechanisms such as activating alternative angiogenic pathways or enhancing invasive capabilities. Furthermore, the primary mechanism of anlotinib leans more towards inhibiting tumor growth (i.e., a cytostatic effect) rather than directly killing tumor cells; consequently, monotherapy often lacks the durable, memory-like disease control that immunotherapy may induce. A study have reported that the anlotinib plus PD-1/PD-L1 inhibitor combination group achieved an mOS of 12.7 months, an mPFS of 8.2 months, and a significantly improved ORR of 57% (41). Compared with monotherapy, the combination regimen demonstrated substantial advantages across all efficacy endpoints, indicating that the combined approach can not only effectively delay tumor progression but also significantly prolong patients’ overall survival.

This marked therapeutic enhancement is underpinned by a well-defined synergistic effect between the two agents (42). Mechanistic investigations have elucidated the multi-faceted basis of this synergy: in a Lewis lung carcinoma (LLC) xenograft model, studies (43) revealed that the combination of anlotinib and PD-1 blockade not only robustly controlled tumor growth but also upregulated chemokine CCL5 expression, thereby efficiently recruiting large numbers of CD8^+^T cells into the tumor core. Notably, specific depletion of CD8^+^T cells abrogated this synergistic effect, directly confirming the central role of CD8^+^T cells in the combination therapy. In another in-depth study (44) the combination of PD-1/PD-L1 blockers and anlotinib was found to promote tumor infiltration of CD4^+^T cells. Specifically, anlotinib downregulates the Janus kinase 2/signal transducer and activator of transcription 3 (JAK2/STAT3) signaling pathway, improved tumor vascular structure and function while also creating favorable conditions for CD4^+^T cell infiltration, thereby significantly enhancing the therapeutic efficacy of PD-1 blockade in the lung cancer model. Collectively, these findings demonstrate that anlotinib optimizes the TME for immune attack through multiple mechanisms: facilitating T cell infiltration (both CD8^+^ and CD4^+^T cells), remodeling aberrant vasculature, regulating the JAK2/STAT3 pathway, and exerting multi-target inhibition, thereby reversing immune resistance at multiple levels. Concurrently, immune checkpoint inhibitors (ICIs) reinvigorate exhausted immune cells within this remodeled TME, enabling them to effectively eliminate tumor cells. Consequently, the combination of anlotinib and ICIs may produce a synergistic antitumor effect. Early preclinical studies have demonstrated that TQB2450 exhibits high binding affinity to human PD-L1 *in vitro*, with a reported affinity of 0.25 nM (45). A first-in-human phase I study demonstrated that TQB2450, a humanized monoclonal antibody targeting PD-L1, simultaneously blocks the binding of PD-L1 to the PD-1 and B7.1 receptors on T cells, thereby restoring T-cell function and potentiating antitumor immunity (46). This dual blockade may result in more comprehensive immune activation. Taken together, these characteristics may contribute to the significantly enhanced efficacy observed when TQB2450 is combined with anlotinib in clinical settings.

In the present study, the combination therapy significantly improved the ORR, particularly in patients with advanced NSCLC and RCC, enabling a higher proportion of patients to achieve tumor shrinkage. It is noteworthy that the pooled DCR across various cancer types was significantly higher than the ORR, a finding of considerable clinical importance. This reveals that the combination regimen’s ability to control tumors is generally superior to its ability to shrink them. In other words, beyond inducing significant tumor shrinkage in a subset of patients, this regimen can achieve long-term disease stabilization in a broader patient population, thereby providing clinical benefit.

In terms of safety, this study found that the toxicity profile of the combination regimen was predictable and generally manageable. In the management of TRAEs, particular attention should be paid to the risk of hypoalbuminemia. Anlotinib may induce hypoalbuminemia through two potential mechanisms: first, by causing liver function damage, which reduces albumin synthesis (47, 48); second, by interfering with podocyte function, disrupting the structure of glomerular endothelial cells, and inducing microangiopathy, ultimately leading to proteinuria and subsequent hypoalbuminemia (49). To effectively manage this risk, the following comprehensive strategy is recommended: strengthen nutritional assessment and support before and during treatment, encourage a high-protein diet, and provide oral nutritional supplements if necessary; monitor serum albumin levels regularly to facilitate early detection and timely intervention. For established hypoalbuminemia, interventions such as dietary reinforcement, oral nutritional supplementation, or medical treatment can be implemented based on severity. These measures, combined with conventional management like antihypertensive therapy and blood count monitoring, can effectively control most known TRAEs associated with anlotinib. Of particular interest is the unique “two weeks on, one week off” dosing schedule of anlotinib, which provides patients with periodic recovery windows (40). This design significantly contributes to managing the cumulative toxicity of the drug, thereby better preserving patients’ quality of life during long-term treatment.

As a multi-targeted agent with broad-spectrum antitumor activity, anlotinib’s application prospects extend far beyond NSCLC. Although its therapeutic role is most established in the field of lung cancer, it is also part of the standard treatment for soft tissue sarcoma, SCLC, and medullary thyroid carcinoma. The value of this study lies in its integrated analysis, which reveals the significant therapeutic potential of this combination regimen across multiple cancer types, including RCC and HCC. These findings suggest that the combination of anlotinib with ICIs holds promise for transcending the limitations of specific cancer types, potentially evolving into a “platform” strategy for cross-cancer therapy. The foundation of this broad-spectrum activity lies in the universality of its mechanism of action-regardless of tumor type, improving the TME and promoting immune cell infiltration are key to enhancing the efficacy of immunotherapy.

Nevertheless, this study has certain limitations. First, the number of included RCTs is limited, and some cancer types are represented by few relevant studies, which restricts the ability to perform more in-depth subgroup analyses. Second, although basic research provides detailed explanations for the synergistic mechanisms of the combination therapy, most of the clinical studies included in this meta-analysis lacked corresponding biomarker analysis results from patient tumor tissues. This creates a disconnect between the clinical outcomes and the mechanistic theory, and the link between mechanism and clinical efficacy requires further confirmation through more integrated basic and clinical research. Third, some included studies received low scores on the NOS quality assessment, which could introduce some bias into the pooled results. Finally, to ensure literature quality, this study only included papers published in major databases such as PubMed, Embase, Cochrane Library, and Web of Science. This may have led to the omission of some negative results published in lower-impact journals, introducing a risk of publication bias.

## 5 Conclusions

In summary, this systematic review and meta-analysis provide compelling evidence for the synergistic antitumor efficacy of combining anlotinib with PD−1/PD−L1 inhibitors across a spectrum of advanced solid tumors, confirming significant improvements in ORR and DCR alongside a substantial prolongation of both PFS and OS. Moving forward, the therapeutic framework warrants further refinement through large−scale phase III trials, the identification of predictive biomarkers such as intratumoral CCL5 expression and T−cell infiltration density, and the optimization of treatment sequencing, dosing, and duration to maximize synergy while preserving a manageable safety profile. Through continued rigorous investigation, this combination strategy is poised to be further optimized, offering renewed potential for durable disease control and improved long−term outcomes for a broader population of cancer patients.

## Data Availability

The original contributions presented in the study are included in the article/**Supplementary Material**. Further inquiries can be directed to the corresponding authors.
