# Correction: Efficacy and safety of anlotinib combined with PD-1/PD-L1 inhibitors in malignant solid tumors: a meta-analysis and network meta-analysis

**DOI:** 10.3389/fimmu.2026.1893679

**Published:** 2026-06-05

**Authors:** Chen Wang, Ning Wang, Zijing Wu, Xinjuan Yu, Xiaolu Yu, Jing Wang, Jun Li, Yaozu Han

**Affiliations:** 1 School of Medicine and Pharmacy, Ocean University of China, Qingdao, China; 2 Department of Respiratory and Critical Care Medicine, Qingdao Municipal Hospital, University of Health and Rehabilitation Sciences, Qingdao, China; 3 Clinical Research Center, Qingdao Key Laboratory of Common Diseases, Qingdao Municipal Hospital, University of Health and Rehabilitation Sciences, Qingdao, China

**Keywords:** anlotinib, malignant solid tumors, meta-analysis, PD-1 inhibitors, PD-L1 inhibitors

Affiliation “^1^School of Medicine and Pharmacy, Ocean University of China, Qingdao, China”, was erroneously given as “Qingdao Key Laboratory of Common Diseases, Qingdao Municipal Hospital, School of Medicine and Pharmacy, Ocean University of China, Qingdao, Shandong, China”.

The original version of this article has been updated.

