# Supplementary material for: Efficacy and safety of anlotinib combined with PD-1/PD-L1 inhibitors in malignant solid tumors: a meta-analysis and network meta-analysis
Source: Front Immunol. 2026 Mar 24;17:1780636. doi: 10.3389/fimmu.2026.1780636 (PMC13055519; doi:10.3389/fimmu.2026.1780636)
Supplement: Supplementary file 1 [file DataSheet1.docx]

# Supplementary Information (SI)

**Efficacy and Safety of Anlotinib Combined with PD-1/PD-L1 Inhibitors in Malignant Solid Tumors: A Meta-Analysis and Network Meta-Analysis**

**This file includes:**

**Supplementary Table 1** Search Strategy.

**Supplementary Table 2** NOS Quality Assessment.

**Supplementary Table 3** Meta-analysis results of TRAEs in randomized and non-randomized controlled studies.

**Supplementary Table 4** Network Meta-Analysis League Table for ORR.

**Supplementary Table 5** Network Meta-Analysis League Table for DCR.

**Supplementary Figure 1** (A) Risk of bias bar chart for individual studies; (B) Risk of bias plot for the pooled effect**.**

Supplementary Figure 2 Subgroup analyses of pooled ORR based on (A) prior treatment status and (B) whether the combination regimen included chemotherapy in RCTs and non-RCTs.

**Supplementary Figure 3** Subgroup analyses of pooled DCR based on (A) prior treatment status and (B) whether the combination regimen included chemotherapy in RCTs and non-RCTs.

**Supplementary Figure 4** ORR Network Plot (A) DCR Network Plot (B) ORR Network Meta-Analysis Forest Plot (C) DCR Network Meta-Analysis Forest Plot (D).

**Supplementary Figure 5** Sensitivity analysis results for ORR in randomized and non-randomized controlled studies(A); Sensitivity analysis results for DCR in randomized and non-randomized controlled studies(B).

Supplementary Figure 6 Sensitivity Analysis Results for ORR in the RCTs(A); Sensitivity Analysis Results for DCR in the RCTs(B).

Supplementary Figure 7 Funnel plot for ORR publication bias in RCTs(A); Funnel plot for DCR publication bias in RCT studies(B).

Supplementary Figure 8 Comparison of ORR - Corrected Funnel Plot (A) Comparison of DCR - Corrected Funnel Plot (B).

Supplementary Table 1 Search Strategy.

| Pubmed | ((((MPDL-3280A[Title/Abstract])) OR (Tecentriq[Title/Abstract])) OR (RG7446[Title/Abstract])) OR (RG-7446[Title/Abstract]))) OR ((cemiplimab[Title/Abstract]) OR (REGN2810[Title/Abstract]))) OR ((((((((((((((((((("Immune Checkpoint Inhibitors"[Mesh]) OR (((((((((((((((((((((((((((((((((Checkpoint Inhibitors, Immune[Title/Abstract]) OR (Immune Checkpoint Blockers[Title/Abstract])) OR (Checkpoint Blockers, Immune[Title/Abstract])) OR (Immune Checkpoint Inhibitor[Title/Abstract])) OR (Checkpoint Inhibitor, Immune[Title/Abstract])) OR (CTLA-4 Inhibitors[Title/Abstract])) OR (CTLA 4 Inhibitors[Title/Abstract])) OR (Cytotoxic T-Lymphocyte-Associated Protein 4 Inhibitors[Title/Abstract])) OR (Cytotoxic T Lymphocyte Associated Protein 4 Inhibitors[Title/Abstract])) OR (Cytotoxic T-Lymphocyte-Associated Protein 4 Inhibitor[Title/Abstract])) OR (Cytotoxic T Lymphocyte Associated Protein 4 Inhibitor[Title/Abstract])) OR (CTLA-4 Inhibitor[Title/Abstract])) OR (CTLA 4 Inhibitor[Title/Abstract])) OR (PD-1 Inhibitors[Title/Abstract])) OR (PD 1 Inhibitors[Title/Abstract])) OR (Programmed Cell Death Protein 1 Inhibitor[Title/Abstract])) OR (Programmed Cell Death Protein 1 Inhibitors[Title/Abstract])) OR (PD-1 Inhibitor[Title/Abstract])) OR (Inhibitor, PD-1[Title/Abstract])) OR (PD 1 Inhibitor[Title/Abstract])) OR (Immune Checkpoint Blockade[Title/Abstract])) OR (Checkpoint Blockade, Immune[Title/Abstract])) OR (Immune Checkpoint Inhibition[Title/Abstract])) OR (Checkpoint Inhibition, Immune[Title/Abstract])) OR (PD-L1 Inhibitors[Title/Abstract])) OR (PD L1 Inhibitors[Title/Abstract])) OR (Programmed Death-Ligand 1 Inhibitors[Title/Abstract])) OR (Programmed Death Ligand 1 Inhibitors[Title/Abstract])) OR (PD-L1 Inhibitor[Title/Abstract])) OR (PD L1 Inhibitor[Title/Abstract])) OR (PD-1-PD-L1 Blockade[Title/Abstract])) OR (Blockade, PD-1-PD-L1[Title/Abstract])) OR (PD 1 PD L1 Blockade[Title/Abstract]))) OR (((((((((tremelimumab[Title/Abstract]) OR (CP-675,206[Title/Abstract])) OR (CP 675206[Title/Abstract])) OR (CP-675206[Title/Abstract])) OR (CP675206[Title/Abstract])) OR (CP 675[Title/Abstract])) OR (CP-675[Title/Abstract])) OR (CP675 cpd[Title/Abstract])) OR (ticilimumab[Title/Abstract]))) OR ((((((((((ipilimumab[Title/Abstract]) OR (Anti-CTLA-4 MAb Ipilimumab[Title/Abstract])) OR (Anti CTLA 4 MAb Ipilimumab[Title/Abstract])) OR (Ipilimumab, Anti-CTLA-4 MAb[Title/Abstract])) OR (MDX 010[Title/Abstract])) OR (MDX-010[Title/Abstract])) OR (MDX010[Title/Abstract])) OR (MDX-CTLA-4[Title/Abstract])) OR (MDX CTLA 4[Title/Abstract])) OR (Yervoy[Title/Abstract]))) OR ((((durvalumab[Title/Abstract]) OR (MEDI4736[Title/Abstract])) OR (MEDI-4736[Title/Abstract])) OR (Imfinzi[Title/Abstract]))) OR ((((((avelumab[Title/Abstract]) OR (MSB0010718C[Title/Abstract])) OR (MSB-0010718C[Title/Abstract])) OR (bavencio[Title/Abstract])) OR (MSB-0010682[Title/Abstract])) OR (MSB0010682[Title/Abstract]))) OR ((((((((atezolizumab[Title/Abstract]) OR (immunoglobulin G1, anti-(human CD antigen CD274) (human monoclonal MDPL3280a heavy chain), disulfide with human monoclonal MDPL3280a kappa-chain, dimer[Title/Abstract])) OR (anti-PDL1[Title/Abstract])) OR (MPDL3280A[Title/Abstract])) OR tislelizumab[Title/Abstract]) OR (tislelizumab-jsgr[Title/Abstract])) OR (BGB-A317[Title/Abstract])) OR (JHL-2108[Title/Abstract])) OR (JHL2108[Title/Abstract])) OR (tevimbra[Title/Abstract]))) OR ((((camrelizumab[Title/Abstract]) OR (SHR-1210[Title/Abstract])) OR (Camrelizumab[Title/Abstract])) OR (SHR 1210[Title/Abstract]))) OR ((((sintilimab[Title/Abstract]) OR (IBI 308[Title/Abstract])) OR (IBI308[Title/Abstract])) OR (IBI-308[Title/Abstract]))) OR (((((pembrolizumab[Title/Abstract]) OR (MK-3475[Title/Abstract])) OR (Keytruda[Title/Abstract])) OR (lambrolizumab[Title/Abstract])) OR (SCH-900475[Title/Abstract]))) OR (toripalimab[Title/Abstract])) OR (((((((((((nivolumab[Title/Abstract]) OR (MDX-1106[Title/Abstract])) OR (MDX1106[Title/Abstract])) OR (MDX 1106[Title/Abstract])) OR (Opdivo[Title/Abstract])) OR (BMS-936558[Title/Abstract])) OR (BMS936558[Title/Abstract])) OR (BMS 936558[Title/Abstract])) OR (ONO-4538[Title/Abstract])) OR (ONO4538[Title/Abstract])) OR (ONO 4538[Title/Abstract]))OR (Cadonilimab[Title/Abstract]))) AND (("Neoplasms"[Mesh]) OR (((((((((((((((((Tumors[Title/Abstract]) OR (Neoplasia[Title/Abstract])) OR (Neoplasias[Title/Abstract])) OR (Neoplasm[Title/Abstract])) OR (Tumor[Title/Abstract])) OR (Cancer[Title/Abstract])) OR (Cancers[Title/Abstract])) OR (Malignant Neoplasm[Title/Abstract])) OR (Malignancy[Title/Abstract])) OR (Malignancies[Title/Abstract])) OR (Malignant Neoplasms[Title/Abstract])) OR (Neoplasm, Malignant[Title/Abstract])) OR (Neoplasms, Malignant[Title/Abstract])) OR (Benign Neoplasms[Title/Abstract])) OR (Neoplasms, Benign[Title/Abstract])) OR (Neoplasm, Benign[Title/Abstract])) OR (Benign Neoplasm[Title/Abstract])))) AND ((Anlotinib[Title/Abstract]) OR (AL3818[Title/Abstract]))) AND (randomized controlled trial [Publication Type] OR randomized [Title/Abstract] OR placebo [Title/Abstract])  **(results:84)** |
| --- | --- |
| Embase | #7. #3 AND #4 AND #5 AND #6 **(results:87)**  #6.'randomized controlled trial':ab,ti OR 'randomized':ab,ti OR 'placebo':ab,ti OR 'rct':ab,ti  #5. 'anlotinib':ab,ti OR 'al3818':ab,ti  #4. 'immune checkpoint inhibitors':ab,ti OR 'checkpoint inhibitors, immune':ab,ti OR 'immune checkpoint blockers':ab,ti OR 'checkpoint blockers, immune':ab,ti OR 'immune checkpoint inhibitor':ab,ti OR 'checkpoint inhibitor, immune':ab,ti OR 'ctla-4 inhibitors':ab,ti OR 'ctla 4 inhibitors':ab,ti OR 'cytotoxic t-lymphocyte-associated protein 4 inhibitors':ab,ti OR 'cytotoxic t lymphocyte associated protein 4 inhibitors':ab,ti OR 'cytotoxic t-lymphocyte-associated protein 4 inhibitor':ab,ti OR 'cytotoxic t lymphocyte associated protein 4 inhibitor':ab,ti OR 'ctla-4 inhibitor':ab,ti OR 'ctla 4 inhibitor':ab,ti OR 'pd-1 inhibitors':ab,ti OR 'pd 1 inhibitors':ab,ti OR 'programmed cell death protein 1 inhibitor':ab,ti OR 'programmed cell death protein 1 inhibitors':ab,ti OR 'pd-1 inhibitor':ab,ti OR 'inhibitor, pd-1':ab,ti OR 'pd 1 inhibitor':ab,ti OR 'immune checkpoint blockade':ab,ti OR 'checkpoint blockade, immune':ab,ti OR 'immune checkpoint inhibition':ab,ti OR 'checkpoint inhibition, immune':ab,ti OR 'pd-l1 inhibitors':ab,ti OR 'pd l1 inhibitors':ab,ti OR 'programmed death-ligand 1 inhibitors':ab,ti OR 'programmed death ligand 1 inhibitors':ab,ti OR 'pd-l1 inhibitor':ab,ti OR 'pd l1 inhibitor':ab,ti OR 'pd-1-pd-l1 blockade':ab,ti OR 'blockade, pd-1-pd-l1':ab,ti OR 'pd 1 pd l1 blockade':ab,ti OR 'pembrolizumab':ab,ti OR 'mk-3475':ab,ti OR 'keytruda':ab,ti OR 'lambrolizumab':ab,ti OR 'sch-900475':ab,ti OR 'nivolumab':ab,ti OR 'mdx-1106':ab,ti OR 'mdx1106':ab,ti OR 'mdx 1106':ab,ti OR 'opdivo':ab,ti OR 'bms-936558':ab,ti OR 'bms936558':ab,ti OR 'bms 936558':ab,ti OR 'ono-4538':ab,ti OR 'ono4538':ab,ti OR 'ono 4538':ab,ti OR 'toripalimab':ab,ti OR 'sintilimab':ab,ti OR 'ibi 308':ab,ti OR 'ibi308':ab,ti OR 'ibi-308':ab,ti OR 'shr-1210':ab,ti OR 'camrelizumab':ab,ti OR 'shr 1210':ab,ti OR 'tislelizumab':ab,ti OR 'tislelizumab-jsgr':ab,ti OR 'bgb-a317':ab,ti OR 'jhl-2108':ab,ti OR 'jhl2108':ab,ti OR 'tevimbra':ab,ti OR 'cemiplimab':ab,ti OR 'regn2810':ab,ti OR 'atezolizumab':ab,ti OR 'immunoglobulin g1, anti-(human cd antigen cd274) (human monoclonal mdpl3280a heavy chain), disulfide with human monoclonal mdpl3280a kappa-chain, dimer':ab,ti OR 'anti-pdl1':ab,ti OR 'mpdl3280a':ab,ti OR 'mpdl-3280a':ab,ti OR 'tecentriq':ab,ti OR 'rg7446':ab,ti OR 'rg-7446':ab,ti OR 'avelumab':ab,ti OR 'msb0010718c':ab,ti OR 'msb-0010718c':ab,ti OR 'bavencio':ab,ti OR 'msb-0010682':ab,ti OR 'msb0010682':ab,ti OR 'durvalumab':ab,ti OR 'medi4736':ab,ti OR 'medi-4736':ab,ti OR 'imfinzi[title':ab,ti OR 'ipilimumab':ab,ti OR 'anti-ctla-4 mab ipilimumab':ab,ti OR 'anti ctla 4 mab ipilimumab':ab,ti OR 'ipilimumab, anti-ctla-4 mab':ab,ti OR 'mdx 010':ab,ti OR 'mdx-010':ab,ti OR 'mdx010':ab,ti OR 'mdx-ctla-4':ab,ti OR 'mdx ctla 4':ab,ti OR 'yervoy':ab,ti OR 'tremelimumab':ab,ti OR 'cp-675,206':ab,ti OR 'cp 675206':ab,ti OR 'cp-675206':ab,ti OR 'cp675206':ab,ti OR 'cp 675':ab,ti OR 'cp-675':ab,ti OR 'cp675 cpd':ab,ti OR 'ticilimumab':ab,ti OR 'cadonilimab':ab,ti  #3. #1 OR #2  #2. 'tumors':ab,ti OR 'neoplasia':ab,ti OR 'neoplasias':ab,ti OR 'neoplasm':ab,ti OR 'tumor':ab,ti OR 'cancer':ab,ti OR 'cancers':ab,ti OR 'malignant neoplasm':ab,ti OR 'malignancy':ab,ti OR 'malignancies':ab,ti OR 'malignant neoplasms':ab,ti OR 'neoplasm, malignant':ab,ti OR 'neoplasms, malignant':ab,ti OR 'benign neoplasms':ab,ti OR 'neoplasms, benign':ab,ti OR 'neoplasm, benign':ab,ti OR 'benign neoplasm':ab,ti  #1. 'neoplasms'/exp OR neoplasms |
| Web  of  sicence | 1: TS=(Neoplasms OR Tumors OR Neoplasia OR Neoplasias OR Neoplasm OR Tumor OR Cancer OR Cancers OR Malignant Neoplasm OR Malignancy OR Malignancies OR Malignant Neoplasms OR Neoplasm, Malignant OR Neoplasms, Malignant OR Benign Neoplasms OR Neoplasms, Benign OR Neoplasm, Benign OR Benign Neoplasm)  2: TS=(Anlotinib OR AL3818)  3: TS=(Immune Checkpoint Inhibitors OR Checkpoint Inhibitors, Immune OR Immune Checkpoint Blockers OR Checkpoint Blockers, Immune OR Immune Checkpoint Inhibitor OR Checkpoint Inhibitor, Immune OR CTLA-4 Inhibitors OR CTLA 4 Inhibitors OR Cytotoxic T-Lymphocyte-Associated Protein 4 Inhibitors OR Cytotoxic T Lymphocyte Associated Protein 4 Inhibitors OR Cytotoxic T-Lymphocyte-Associated Protein 4 Inhibitor OR Cytotoxic T Lymphocyte Associated Protein 4 Inhibitor OR CTLA-4 Inhibitor OR CTLA 4 Inhibitor OR PD-1 Inhibitors OR PD 1 Inhibitors OR Programmed Cell Death Protein 1 Inhibitor OR Programmed Cell Death Protein 1 Inhibitors OR PD-1 Inhibitor OR Inhibitor, PD-1 OR PD 1 Inhibitor OR Immune Checkpoint Blockade OR Checkpoint Blockade, Immune OR Immune Checkpoint Inhibition OR Checkpoint Inhibition, Immune OR PD-L1 Inhibitors OR PD L1 Inhibitors OR Programmed Death-Ligand 1 Inhibitors OR Programmed Death Ligand 1 Inhibitors OR PD-L1 Inhibitor OR PD L1 Inhibitor OR PD-1-PD-L1 Blockade OR Blockade, PD-1-PD-L1 OR PD 1 PD L1 Blockade OR pembrolizumab OR MK-3475 OR Keytruda OR lambrolizumab OR SCH-900475 OR nivolumab OR MDX-1106 OR MDX1106 OR MDX 1106 OR Opdivo OR BMS-936558 OR BMS936558 OR BMS 936558 OR ONO-4538 OR ONO4538 OR ONO 4538 OR toripalimab OR sintilimab OR IBI 308 OR IBI308 OR IBI-308 OR camrelizumab OR SHR-1210 OR Camrelizumab OR SHR 1210 OR tislelizumab OR tislelizumab-jsgr OR BGB-A317 OR JHL-2108 OR JHL2108 OR tevimbra OR cemiplimab OR REGN2810 OR atezolizumab OR immunoglobulin G1, anti-(human CD antigen CD274) (human monoclonal MDPL3280a heavy chain), disulfide with human monoclonal MDPL3280a kappa-chain, dimer OR anti-PDL1 OR MPDL3280A OR MPDL-3280A OR Tecentriq OR RG7446 OR RG-7446 OR avelumab OR MSB0010718C OR MSB-0010718C OR bavencio OR MSB-0010682 OR MSB0010682 OR durvalumab OR MEDI4736 OR MEDI-4736 OR Imfinzi[Title OR ipilimumab OR Anti-CTLA-4 MAb Ipilimumab OR Anti CTLA 4 MAb Ipilimumab OR Ipilimumab, Anti-CTLA-4 MAb OR MDX 010 OR MDX-010 OR MDX010 OR MDX-CTLA-4 OR MDX CTLA 4 OR Yervoy OR tremelimumab OR CP-675,206 OR CP 675206 OR CP-675206 OR CP675206 OR CP 675 OR CP-675 OR CP675 cpd OR ticilimumab OR Cadonilimab)  4: TS=(randomized controlled trial OR randomized OR placebo OR RCT)  5: #1 AND #2 AND #3 AND #4 **(results:55)** |
| Cochrane Library | #1 Neoplasms  #2 (Tumors):ab,ti,kw OR (Neoplasia):ab,ti,kw OR (Neoplasias):ab,ti,kw OR (Neoplasm):ab,ti,kw OR (Tumor):ab,ti,kw OR (Cancer):ab,ti,kw OR (Cancers):ab,ti,kw OR (Malignant Neoplasm):ab,ti,kw OR (Malignancy):ab,ti,kw OR (Malignancies):ab,ti,kw OR (Malignant Neoplasms):ab,ti,kw OR (Neoplasm, Malignant):ab,ti,kw OR (Neoplasms, Malignant):ab,ti,kw OR (Benign Neoplasms):ab,ti,kw OR (Neoplasms, Benign):ab,ti,kw OR (Neoplasm, Benign):ab,ti,kw OR (Benign Neoplasm):ab,ti,kw 275836  #3 #1 OR #2  #4 (Anlotinib):ab,ti,kw OR (AL3818):ab,ti,kw  #5 (Immune Checkpoint Inhibitors):ab,ti,kw OR (Checkpoint Inhibitors, Immune):ab,ti,kw OR (Immune Checkpoint Blockers):ab,ti,kw OR (Checkpoint Blockers, Immune):ab,ti,kw OR (Immune Checkpoint Inhibitor):ab,ti,kw OR (Checkpoint Inhibitor, Immune):ab,ti,kw OR (CTLA-4 Inhibitors):ab,ti,kw OR (CTLA 4 Inhibitors):ab,ti,kw OR (Cytotoxic T-Lymphocyte-Associated Protein 4 Inhibitors):ab,ti,kw OR (Cytotoxic T Lymphocyte Associated Protein 4 Inhibitors):ab,ti,kw OR (Cytotoxic T-Lymphocyte-Associated Protein 4 Inhibitor):ab,ti,kw OR (Cytotoxic T Lymphocyte Associated Protein 4 Inhibitor):ab,ti,kw OR (CTLA-4 Inhibitor):ab,ti,kw OR (CTLA 4 Inhibitor):ab,ti,kw OR (PD-1 Inhibitors):ab,ti,kw OR (PD 1 Inhibitors):ab,ti,kw OR (Programmed Cell Death Protein 1 Inhibitor):ab,ti,kw OR (Programmed Cell Death Protein 1 Inhibitors):ab,ti,kw OR (PD-1 Inhibitor):ab,ti,kw OR (Inhibitor, PD-1):ab,ti,kw OR (PD 1 Inhibitor):ab,ti,kw OR (Immune Checkpoint Blockade):ab,ti,kw OR (Checkpoint Blockade, Immune):ab,ti,kw OR (Immune Checkpoint Inhibition):ab,ti,kw OR (Checkpoint Inhibition, Immune):ab,ti,kw OR (PD-L1 Inhibitors):ab,ti,kw OR (PD L1 Inhibitors):ab,ti,kw OR (Programmed Death-Ligand 1 Inhibitors):ab,ti,kw OR (Programmed Death Ligand 1 Inhibitors):ab,ti,kw OR (PD-L1 Inhibitor):ab,ti,kw OR (PD L1 Inhibitor):ab,ti,kw OR (pembrolizumab):ab,ti,kw OR (MK-3475):ab,ti,kw OR (Keytruda):ab,ti,kw OR (lambrolizumab):ab,ti,kw OR (SCH-900475):ab,ti,kw OR (nivolumab):ab,ti,kw OR (Opdivo):ab,ti,kw OR (BMS-936558):ab,ti,kw OR (BMS936558):ab,ti,kw OR (BMS 936558):ab,ti,kw OR (ONO-4538):ab,ti,kw OR (ONO4538):ab,ti,kw OR (ONO 4538):ab,ti,kw OR (toripalimab):ab,ti,kw OR (sintilimab):ab,ti,kw OR (IBI 308):ab,ti,kw OR (IBI308):ab,ti,kw OR (IBI-308):ab,ti,kw OR (camrelizumab):ab,ti,kw OR (SHR-1210):ab,ti,kw OR (Camrelizumab):ab,ti,kw OR (SHR 1210):ab,ti,kw OR (tislelizumab):ab,ti,kw OR (tislelizumab-jsgr):ab,ti,kw OR (BGB-A317):ab,ti,kw OR (JHL-2108):ab,ti,kw OR (JHL2108):ab,ti,kw OR (tevimbra):ab,ti,kw OR (cemiplimab):ab,ti,kw OR (REGN2810):ab,ti,kw OR (atezolizumab):ab,ti,kw OR (immunoglobulin G1, anti-(human CD antigen CD274) (human monoclonal MDPL3280a heavy chain), disulfide with human monoclonal MDPL3280a kappa-chain, dimer):ab,ti,kw OR (anti-PDL1):ab,ti,kw OR (MPDL3280A):ab,ti,kw OR (MPDL-3280A):ab,ti,kw OR (Tecentriq):ab,ti,kw OR (RG7446):ab,ti,kw OR (RG-7446):ab,ti,kw OR (avelumab):ab,ti,kw OR (MSB0010718C):ab,ti,kw OR (MSB-0010718C):ab,ti,kw OR (bavencio):ab,ti,kw OR (MSB-0010682):ab,ti,kw OR (MSB0010682):ab,ti,kw OR (durvalumab):ab,ti,kw OR (MEDI4736):ab,ti,kw OR (MEDI-4736):ab,ti,kw OR (Imfinzi):ab,ti,kw OR (ipilimumab):ab,ti,kw OR (tremelimumab):ab,ti,kw OR (ticilimumab):ab,ti,kw OR (Cadonilimab):ab,ti,kw  #6 (randomized controlled trial ):ab,ti,kw OR (randomized ):ab,ti,kw OR (placebo):ab,ti,kw OR (RCT ):ab,ti,kw  #7 #3 AND #4 AND #5 AND #6 **(results:75)** |

Supplementary Table 2 NOS Quality Assessment.

| Study | Selection | Comparability | Exposure | Score |
| --- | --- | --- | --- | --- |
| B. Han et al. 2021 | * | ** | ** | 5 |
| C. Han et al. 2022 | ** | ** | ** | 6 |
| C. Lan et al. 2022 | *** | ** | *** | 8 |
| Q. Xu et al. 2022 | *** | ** | ** | 7 |
| S. Jin et al. 2023 | *** | ** | *** | 8 |
| N. Yang et al. 2023 | ** | ** | ** | 6 |
| Y. Zhang et al. 2023 | ** | ** | ** | 6 |
| H. Yuan et al. 2025 | *** | ** | ** | 7 |
| Y. Liu et al. 2022 | *** | ** | *** | 8 |
| W. Zhang et al. 2024 | *** | ** | *** | 8 |
| S. Ma et al. 2024 | *** | ** | *** | 8 |
| X. Meng et al. 2025 | *** | ** | *** | 8 |

Supplementary Table 3 Meta-analysis results of TRAEs in randomized and non-randomized controlled studies.

| Adverse events | Study | Heterogeneity | | ES (95%CI) | P |
| --- | --- | --- | --- | --- | --- |
|  |  | P | I^2^ |  |  |
| Anemia | 12 | P < 0.001 | 96.76 | 0.30(0.14,0.49) | P < 0.001 |
| Hypertension | 13 | P < 0.001 | 93.42 | 0.41(0.28,0.54) | P < 0.001 |
| Reduced platelet count | 13 | P < 0.001 | 97.5 | 0.30(0.13,0.51) | P < 0.001 |
| AST elevation | 11 | P < 0.001 | 84.72 | 0.34(0.25,0.43) | P < 0.001 |
| Fatigue | 11 | P < 0.001 | 83.52 | 0.33(0.25,0.43) | P < 0.001 |
| Hand-foot skin reaction | 10 | P < 0.001 | 94.07 | 0.22(0.11,0.35) | P < 0.001 |
| Proteinuria | 11 | P < 0.001 | 94.18 | 0.24(0.12,0.38) | P < 0.001 |
| Diarrhea | 11 | P < 0.001 | 84.07 | 0.18(0.11,0.26) | P < 0.001 |
| Hypoalbuminemia | 10 | P < 0.001 | 88.32 | 0.34(0.24,0.44) | P < 0.001 |
| Reduced neutrophil | 9 | P < 0.001 | 98.56 | 0.32(0.08,0.63) | P < 0.001 |
| Nausea | 11 | P < 0.001 | 93.13 | 0.20(0.10,0.32) | P < 0.001 |

Supplementary Table 4 Network Meta-Analysis League Table for ORR.

| ANTN+TQB2450 | 0.91 (0.43,1.91) | 0.86 (0.39,1.90) | 0.55 (0.36,0.82) |
| --- | --- | --- | --- |
| 1.10 (0.52,2.31) | ANTN+SIN | 0.95 (0.38,2.37) | 0.60 (0.32,1.12) |
| 1.16 (0.53,2.56) | 1.06 (0.42,2.65) | ANTN+CAM | 0.64 (0.32,1.25) |
| 1.83 (1.21,2.76) | 1.66 (0.89,3.11) | 1.57 (0.80,3.10) | Control |

Supplementary Table 5 Network Meta-Analysis League Table for DCR.

| ANTN+TQB2450 | 1.00 (0.88,1.13) | 0.97 (0.86,1.08) | 0.92 (0.86,0.99) |
| --- | --- | --- | --- |
| 1.00 (0.88,1.14) | ANTN+SIN | 0.97 (0.84,1.12) | 0.92 (0.82,1.03) |
| 1.03 (0.92,1.16) | 1.03 (0.89,1.19) | ANTN+CAM | 0.95 (0.87,1.04) |
| 1.09 (1.01,1.17) | 1.08 (0.97,1.22) | 1.05 (0.96,1.15) | Control |


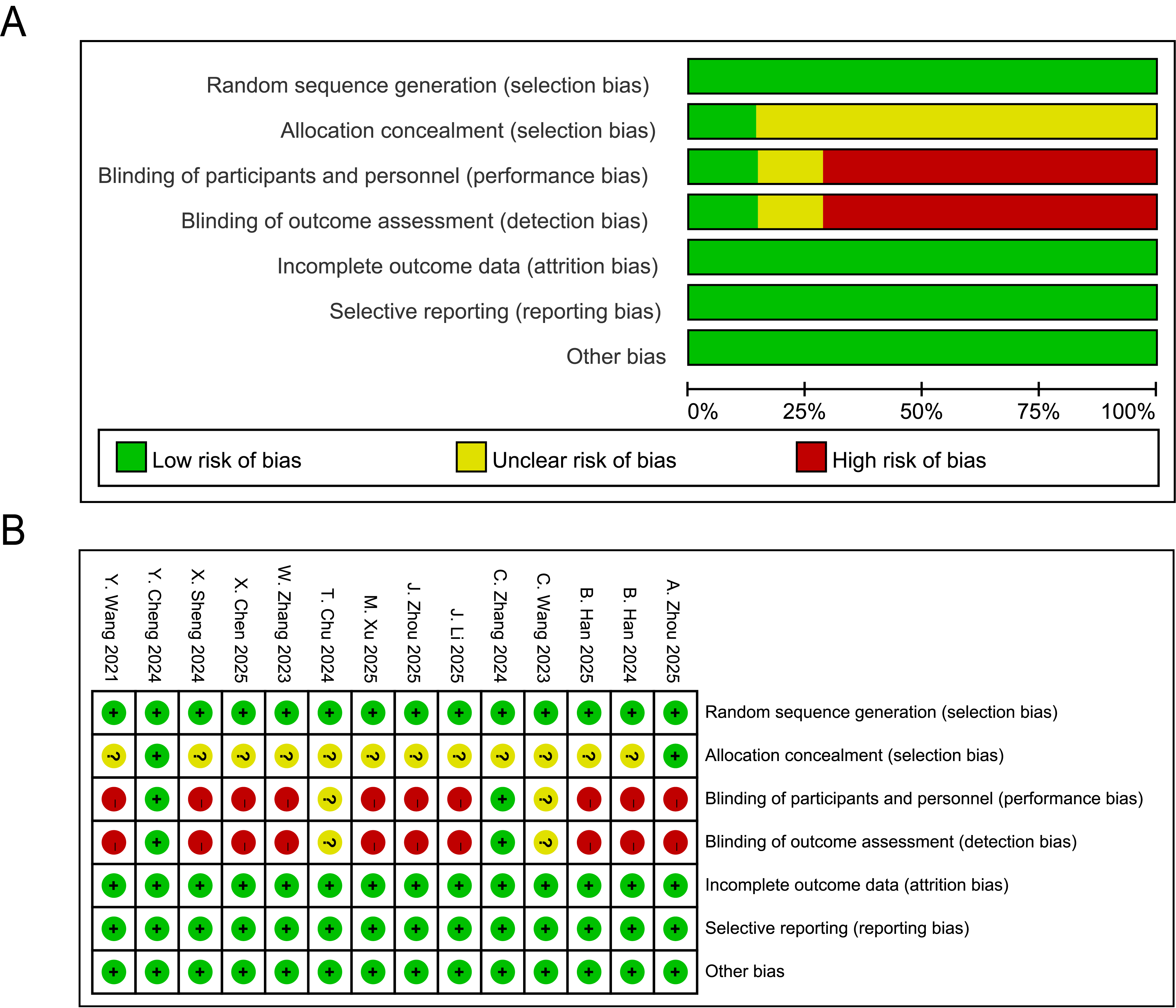


Supplementary Figure 1 (A) Risk of bias bar chart for individual studies; (B) Risk of bias plot for the pooled effect.


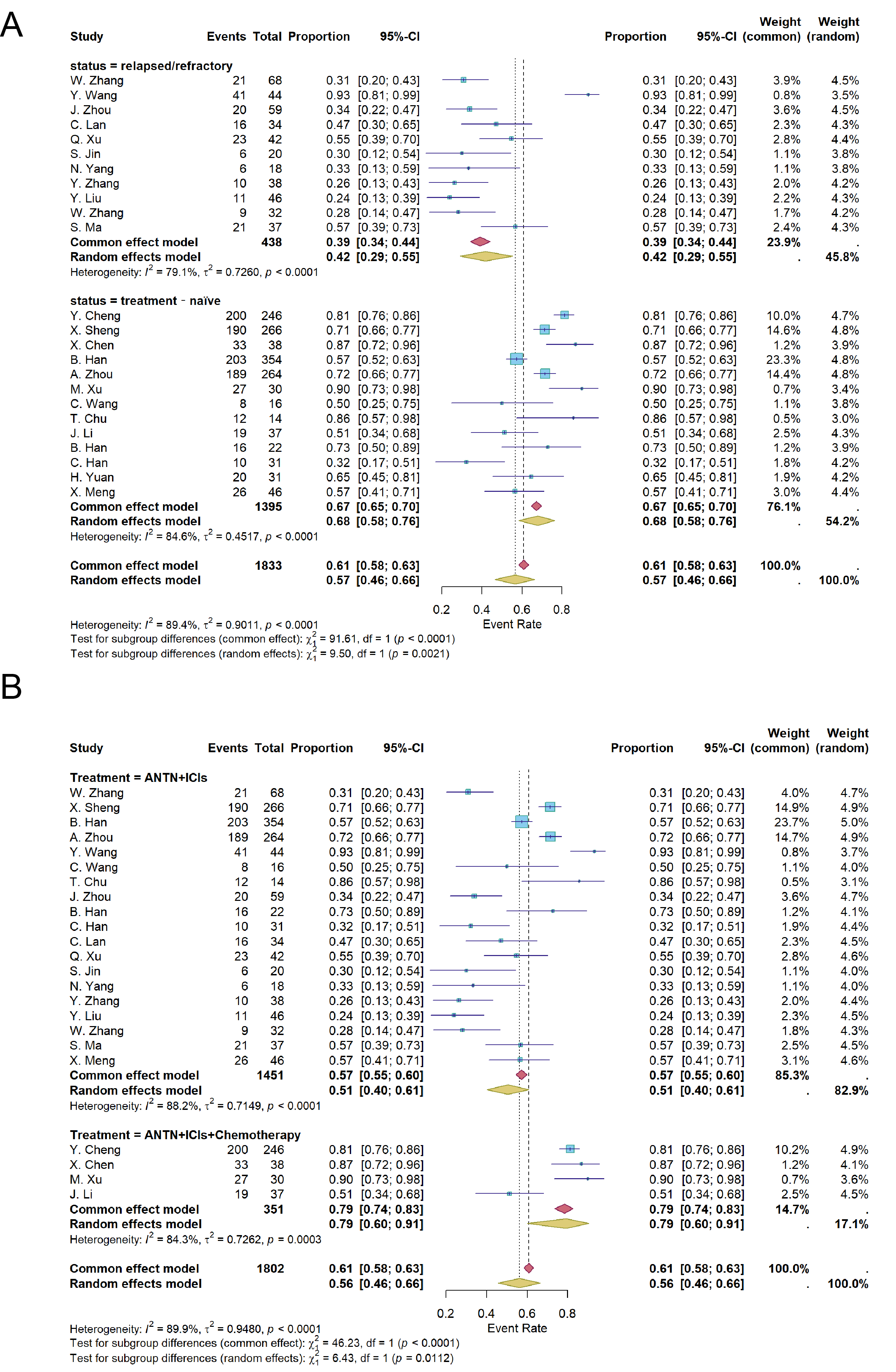


Supplementary Figure 2 Subgroup analyses of pooled ORR based on (A) prior treatment status and (B) whether the combination regimen included chemotherapy in RCTs and non-RCTs.


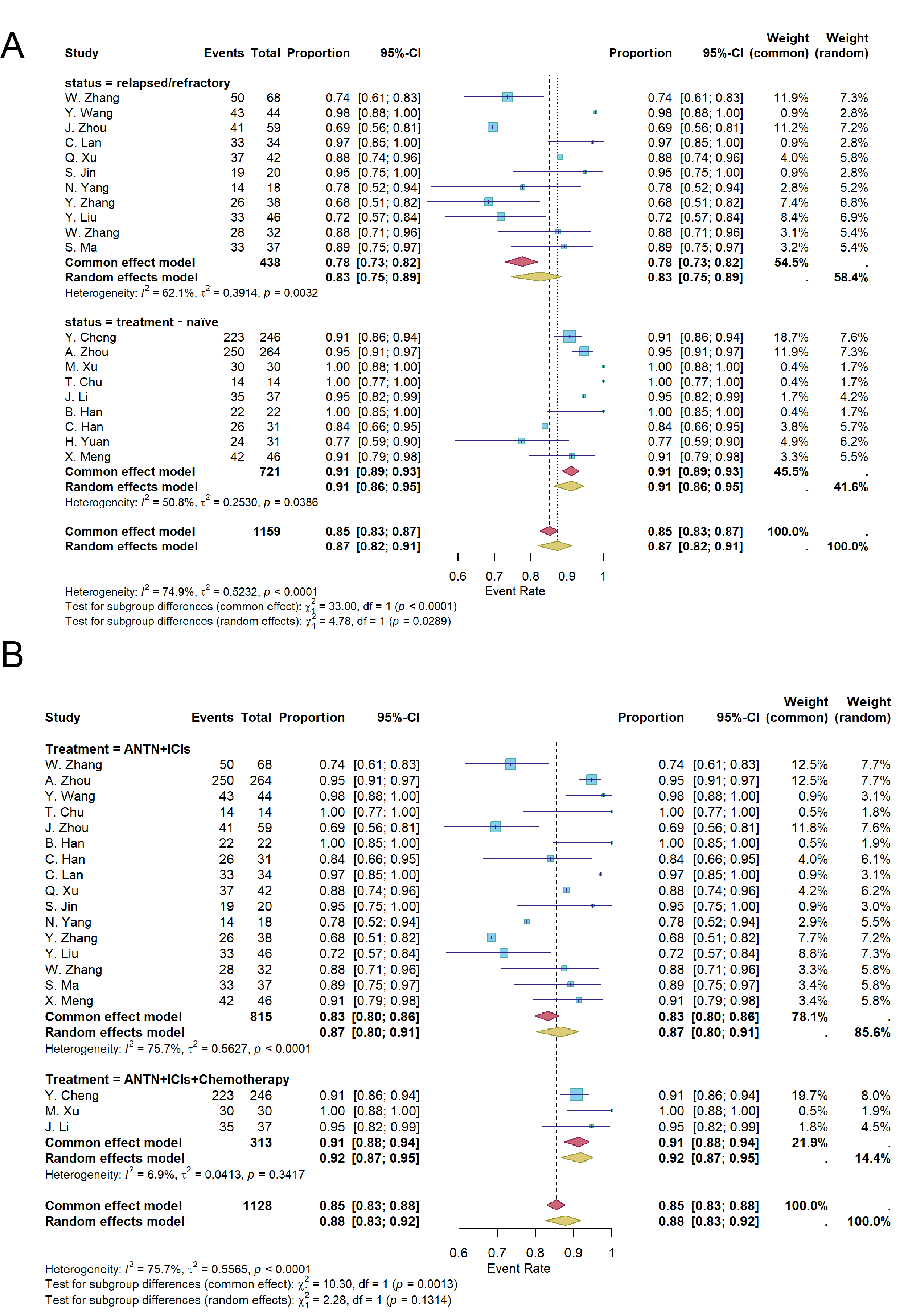


Supplementary Figure 3 Subgroup analyses of pooled DCR based on (A) prior treatment status and (B) whether the combination regimen included chemotherapy in RCTs and non-RCTs.


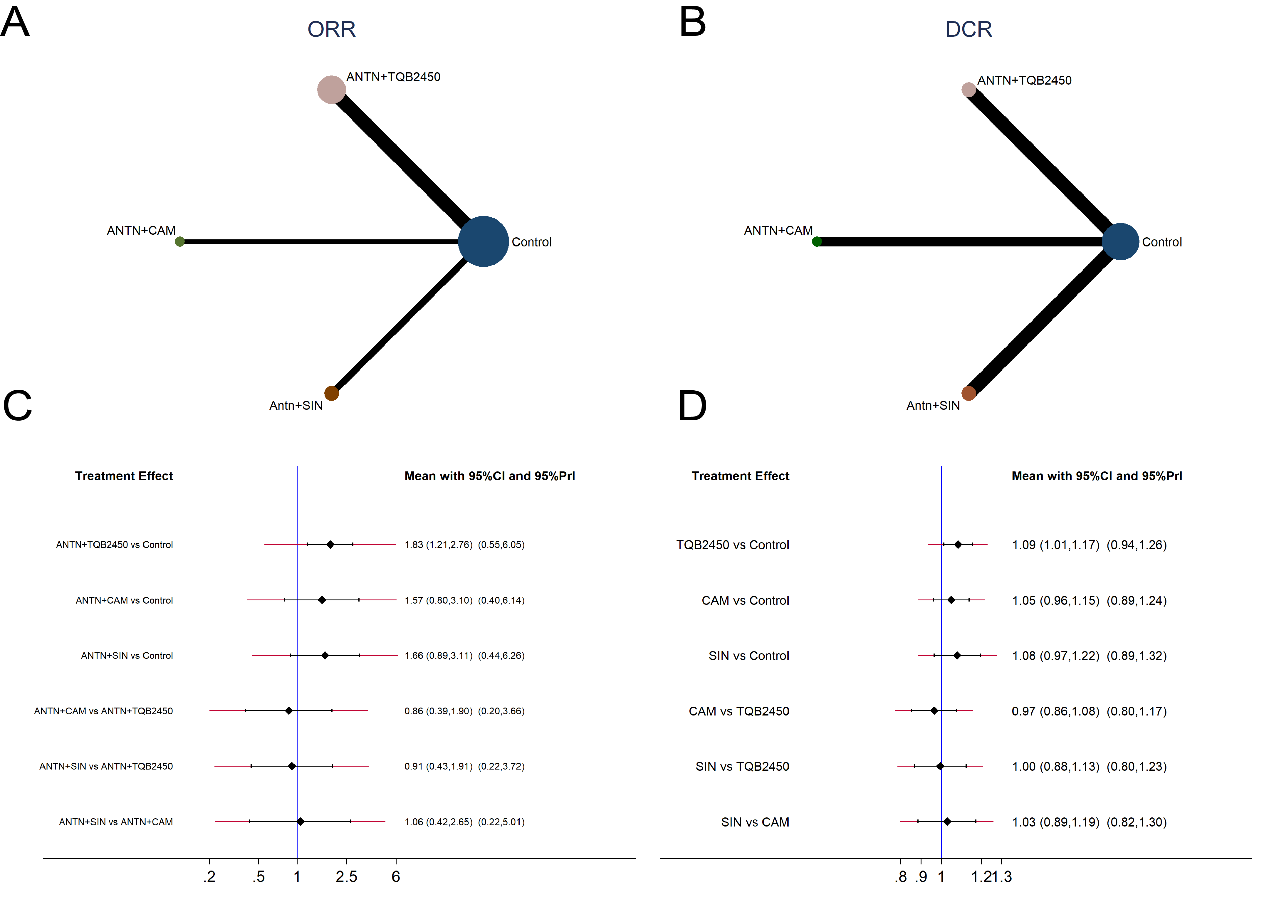


Supplementary Figure 4 ORR Network Plot (A) DCR Network Plot (B) ORR Network Meta-Analysis Forest Plot (C) DCR Network Meta-Analysis Forest Plot (D).


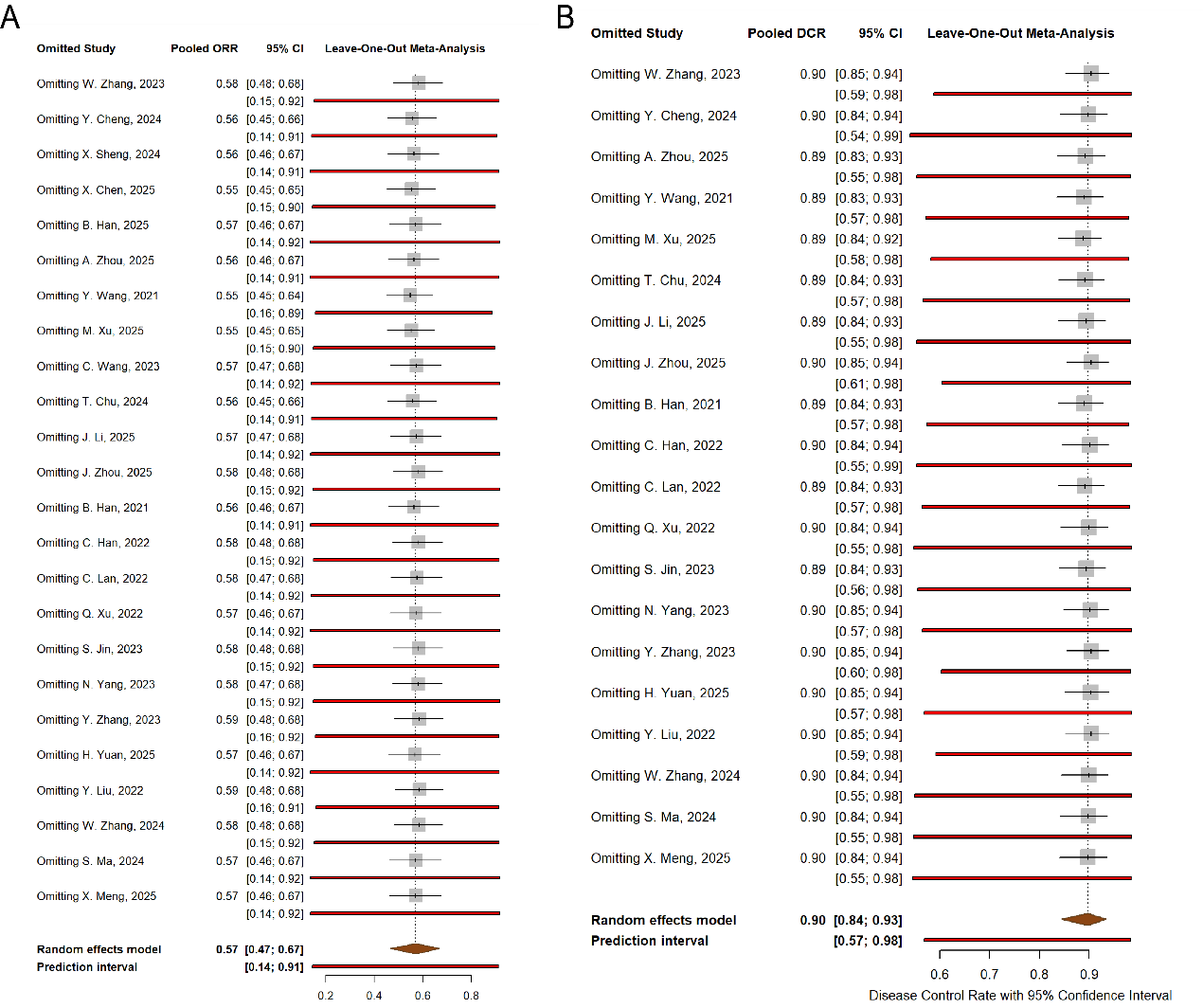


Supplementary Figure 5 Sensitivity analysis results for ORR in randomized and non-randomized controlled studies(A); Sensitivity analysis results for DCR in randomized and non-randomized controlled studies(B).


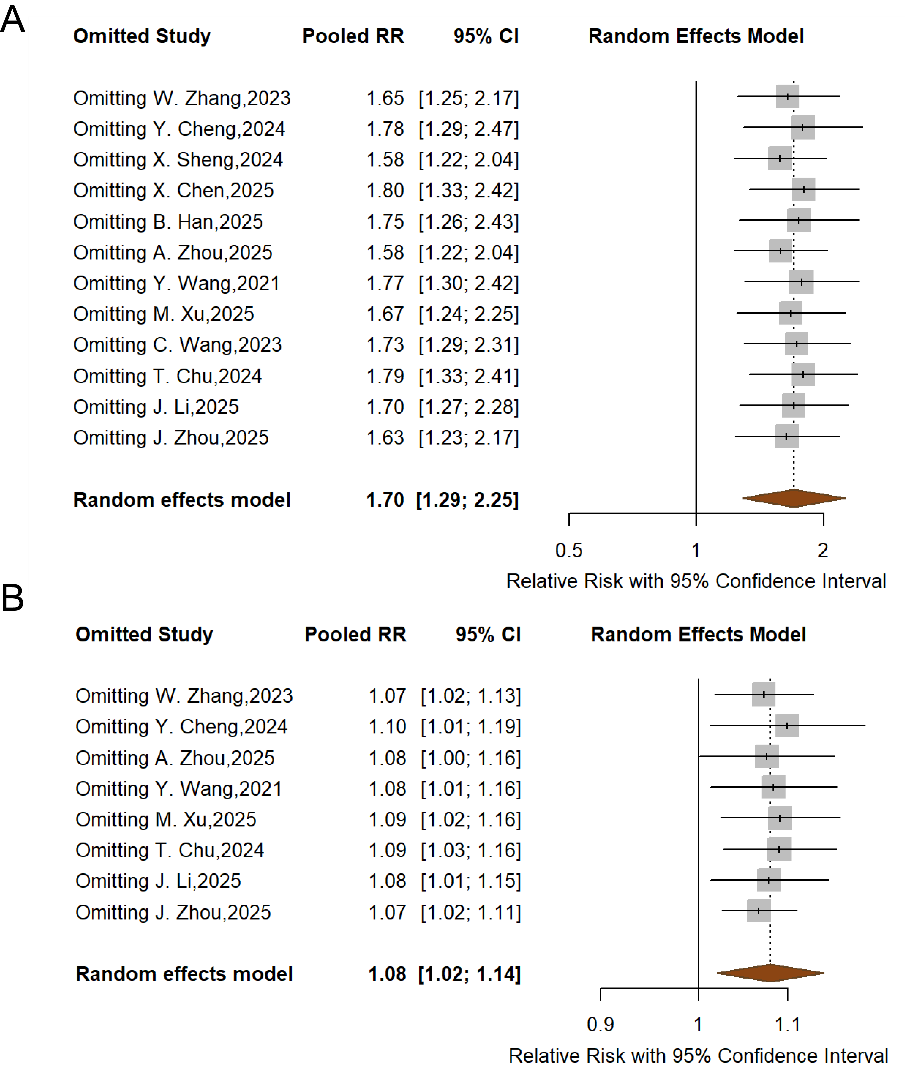


Supplementary Figure 6 Sensitivity Analysis Results for ORR in the RCTs(A); Sensitivity Analysis Results for DCR in the RCTs(B).


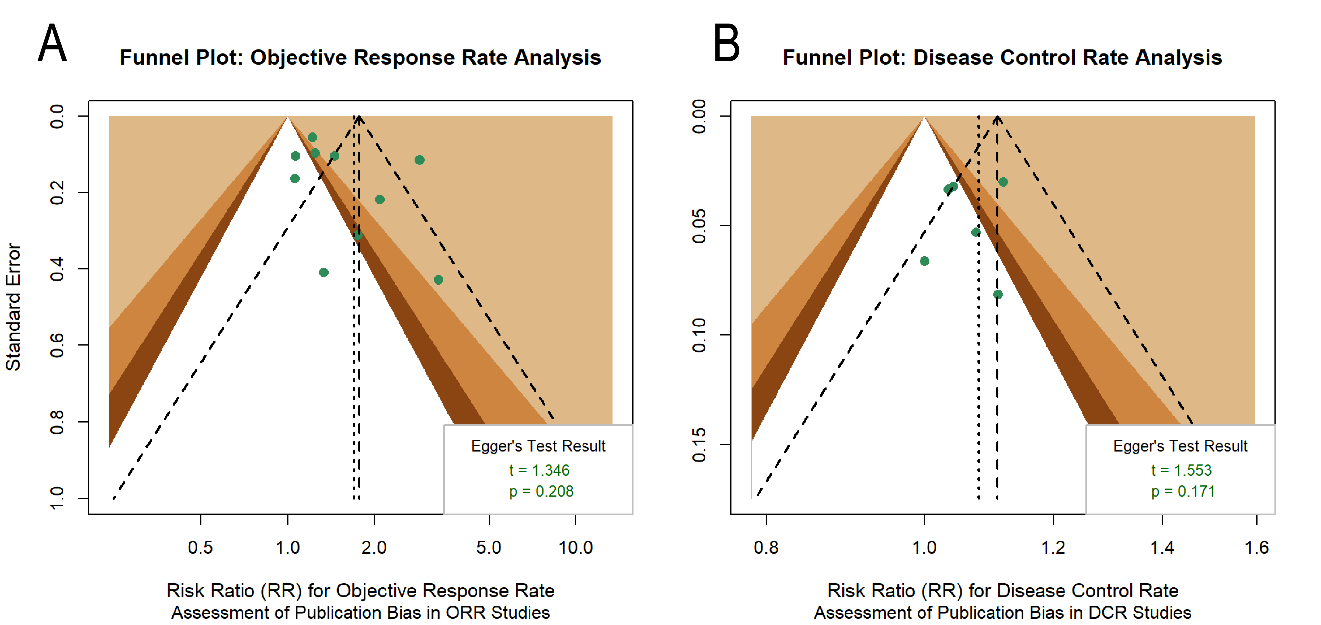


Supplementary Figure 7 Funnel plot for ORR publication bias in RCTs(A);Funnel plot for DCR publication bias in RCT studies(B).
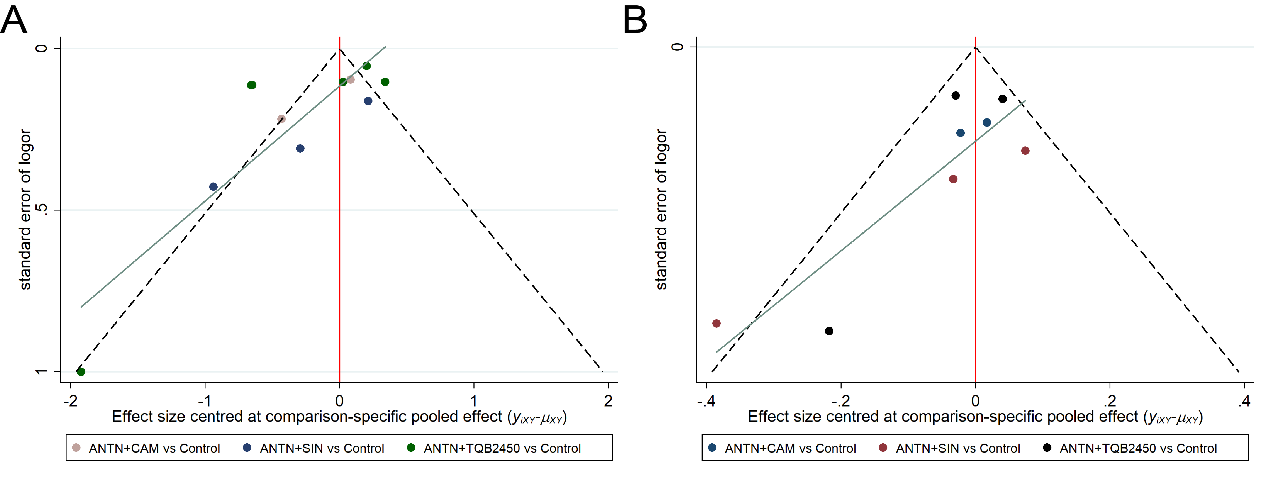
Supplementary Figure 8 Comparison of ORR - Corrected Funnel Plot (A) Comparison of DCR - Corrected Funnel Plot (B).
